# Skeletal muscle-derived interstitial progenitor cells (PICs) display stem cell properties, being clonogenic, self-renewing, and multi-potent in vitro and in vivo

**DOI:** 10.1186/s13287-017-0612-4

**Published:** 2017-07-04

**Authors:** Beverley J. Cottle, Fiona C. Lewis, Victoria Shone, Georgina M. Ellison-Hughes

**Affiliations:** Centre of Human & Aerospace Physiological Sciences & Centre for Stem Cells and Regenerative Medicine, Faculty of Life Sciences & Medicine, King’s College London, Shepherd’s House, Rm 4.16, Guy’s Campus, London, SE1 1UL UK

**Keywords:** PIC, PW1, Sca-1, Muscle-derived, Stem progenitor cell, Multipotent, Clonogenic, Cell culture

## Abstract

**Background:**

The development of cellular therapies to treat muscle wastage with disease or age is paramount. Resident muscle satellite cells are not currently regarded as a viable cell source due to their limited migration and growth capability ex vivo. This study investigated the potential of muscle-derived PW1^+^/Pax7^–^ interstitial progenitor cells (PICs) as a source of tissue-specific stem/progenitor cells with stem cell properties and multipotency.

**Methods:**

Sca-1^+^/PW1^+^ PICs were identified on tissue sections from hind limb muscle of 21-day-old mice, isolated by magnetic-activated cell sorting (MACS) technology and their phenotype and characteristics assessed over time in culture. Green fluorescent protein (GFP)-labelled PICs were used to determine multipotency in vivo in a tumour formation assay.

**Results:**

Isolated PICs expressed markers of pluripotency (Oct3/4, Sox2, and Nanog), were clonogenic, and self-renewing with >60 population doublings, and a population doubling time of 15.8 ± 2.9 h. PICs demonstrated an ability to generate both striated and smooth muscle, whilst also displaying the potential to differentiate into cell types of the three germ layers both in vitro and in vivo. Moreover, PICs did not form tumours in vivo.

**Conclusion:**

These findings open new avenues for a variety of solid tissue engineering and regeneration approaches, utilising a single multipotent stem cell type isolated from an easily accessible source such as skeletal muscle.

**Electronic supplementary material:**

The online version of this article (doi:10.1186/s13287-017-0612-4) contains supplementary material, which is available to authorized users.

## Background

Muscular dystrophy results in progressive skeletal muscle weakness and wastage [1] due to defects and/or the inability to make the protein dystrophin and other associated protein complexes. There are approximately 70,000 babies, children, and adults in the UK suffering from some form of muscular disease, with 12.6% of these being dystrophic disorders such as Duchenne’s muscular dystrophy (DMD). DMD is a progressive muscle wasting disease which affects approximately 1 in 3500 boys and often results in death due to respiratory failure before 25 years of age [2]. Current treatments include physiotherapy and corticosteroids to improve muscle strength, along with surgery to correct associated spinal curvature; these treatments, however, are not a cure and only serve to manage the condition. Muscle degeneration also occurs with ageing (termed sarcopenia) where the body’s ability to replace muscle fibres decreases resulting in a progressive loss of muscle mass with a reduction in the cross-sectional area of muscle fibres [3]. Indeed, 20% of all 60–70 year olds have sarcopenia, and this figure rises to 50% in those over 75 years [4].

Stem cell regenerative cellular therapies may eventually provide effective treatments for the restoration of skeletal muscle weakness and wastage. In conditions such as muscular dystrophy or sarcopenia, where the condition affects a large area and important tissue of the human body, it is imperative that we develop cellular therapies that can repair damage and restore function in situ [5]. Skeletal muscle harbours resident progenitor satellite cells which are capable of myogenic differentiation in vitro and in vivo and extensively contribute to new muscle fibre formation [6]. However, transplanted satellite cells have limited migration capacity with an inability to cross the endothelial wall [7, 8]. Satellite cells that have undergone culture ex vivo also have decreased proliferative capacity and a reduction in myofibre production when transplanted in vivo [9, 10]; therefore, satellite cells are not currently regarded as a viable source for transplantation regenerative cell therapies.

Since the first discovery of satellite cells 50 years ago [11], a variety of other muscle-derived stem cells have been identified. These include vessel-associated cells such as mesoangioblasts [12] and pericytes [13, 14], alongside interstitial cells such as side population cells [15], fibro/adipogenic progenitors (FAPs) [16], and PW1-positive interstitial cells (PICs) [17]. PW1^+^/Pax7^–^ PICs are bi-potent, efficiently contributing to smooth and skeletal muscle regeneration in vivo, whilst also generating satellite cells and replenishing the PIC population [17]. PICs have also been described by us in the hind limb muscle of pigs [18]. PW1 (also known as paternally expressed gene 3, or peg3) is a nuclear protein expressed in both PICs and quiescent satellite cells, although it is not known if activated satellite cells continue to express PW1 [17, 18]. PICs and satellite cells are distinguishable by both location and molecular markers; satellite cells express Pax7 and are located under the basal lamina [11], whilst PICs do not express Pax7 and are located in the interstitium [17, 18]. Murine PICs are also positive for stem cell antigen (Sca)-1, whilst both satellite cells and PICs are negative for CD45. Approximately 15% of myoblasts also express PW1, most commonly during the final stages of mitosis [19]. Transcriptome and gene ontology analyses of PICs and satellite cells from 1-week-old mice show that both cell types have distinct transcriptome signatures, with satellite cell-specific genes belonging primarily to the skeletal muscle lineage, whilst PICs express genes from multiple cell fates [20]. Utilising PW1-reporter transgenic mice, PW1 expression has been described in multiple adult stem cell niches throughout the body including the gut, skin, central nervous system (CNS), and early haematopoietic stem cells [21]. Therefore, it has been implicated as a possible marker for stem/progenitor cell populations throughout the adult mammalian body.

One of the bottlenecks with the translation of stem cell therapy to the clinic is the generation of enough stem/progenitor cells that maintain their phenotype and differentiation potential to produce efficacious results in vivo. Results of recent clinical trials where bone marrow-derived cells are transplanted after myocardial infarction (MI) have been disappointing [22, 23]. This is due to low cell survival and retention, a hostile host environment, and subsequent restriction of cell proliferation, integration, and differentiation [24–26]. The majority of the trials performed to date have utilised freshly isolated unfractionated bone marrow to avoid introducing variability associated with expanding these cells in vitro [27].

Stem cells are maintained in a quiescent state until activated by injury or another stimulus [28, 29]. Therefore, a freshly isolated stem cell is quiescent and, unless activated in vitro, will not exit from G0 and upon transplantation, coupled with the hostile host environment, will be more prone to death. Therefore, to maximise their survival and regenerative potential, stem/progenitor cells need to be propagated in vitro to large numbers before transplantation in vivo. Therefore, the in vitro propagation and characterisation of the cells over culture time needs to be optimised and validated. Moreover, the use of a clonogenic cell population, which is a homogeneous and uniform therapeutic product, would mean patients receive the same dose of identical cells and the only independent variable would be the patients themselves. Here we assessed whether PW1^+^/Pax7^–^ PICs behave as tissue-specific stem/progenitor cells by exhibiting properties of clonogenicity, self-renewal, and multi-potency in vitro and in vivo.

## Methods

### Animals

All experimental procedures were performed in accordance with the British Home Office Animals (Scientific Procedures) Act 1986 by appropriately qualified staff and approved by the institutional animal welfare and ethical review board of King’s College London. C57BL/6 mice were sacrificed at 3 days (2.6 ± 0.1 g), 10 days (5.4 ± 0.2 g), 21 days (7.8 ± 0.4 g), and 2 years (35.7 ± 1.2 g) of age via CO_2_ asphyxiation or cervical dislocation.

### Immunohistochemistry/immunocytochemistry

Immediately after sacrifice dissected skeletal hind limb muscle was fixed in formalin for 24 h before tissue processing and embedding in paraffin wax blocks. Tissue sections (5 μm) were cut and mounted on polysine™ microscope slides. Following deparaffinisation, rehydration, and antigen retrieval, tissue sections were blocked with 10% donkey serum.

Cells grown on chamber slides were fixed with 4% formaldehyde for 20 min at room temperature.

For cytospin preparations, 10,000 cells per spot were spun down onto polysine™ slides using a Cytospin 4 centrifuge and Shandon EZ double cytofunnels, then fixed with Shandon cellfix spray. The fixative was removed with 95% ethanol for 15 min. For staining of nucleic proteins, cells were permeabilised with 0.1% triton before cells were blocked with 10% donkey serum in 0.1% Tween phosphate-buffered saline (PBS).

Primary antibodies were applied for either 1 h at 37 °C or overnight at 4 °C (See Additional file 1: Table S1 for a list of antibodies). Relevant secondary antibodies were applied (1/100 dilution) for 1 h at 37 °C. Nuclei were counterstained with the DNA binding dye 4,6-diamidino-2-phenylindole (DAPI; 1:1000) for 15 min at room temperature.

All quantification was conducted at × 40 magnification on a fluorescence microscope (Nikon, E1000M Eclipse) and representative Z stack micrographs imaged on a confocal microscope (Zeiss, LSM 710).

### Haematoxylin and eosin staining

To visualise different cell types within the tumour, sections were stained with haematoxylin and eosin to identify nuclei and cytoplasm respectively.

### DAB staining

To determine which tumour cells arose from GFP^+^ PICs and were not a result of the embryonic stem cells (ESCs), paraffin-embedded tumour tissue sections were prepared and stained for green fluorescent protein (GFP; Abcam; 1 h at 37 °C) and endogenous peroxidase activity was blocked using 1 part H_2_O_2_ to 3 parts PBS for 15 min at room temperature. Donkey-anti-Rabbit HRP (Santa Cruz, Texas, USA) was applied for 1 h at 37 °C and SigmaFast DAB tablets were used to visualise the GFP.

### Cell isolation

Skeletal muscle hind limb from 21-day-old mice was dissected and rinsed in basic buffer (MEM, 2.93 mM Hepes, 2.05 mM glutamine, 9.99 mM taurine, pH 7.3). The tissue was minced extensively with scissors and stirred at 37 °C for 30 min in digestion buffer (basic buffer (as above), 7 mg/ml collagenase II). Following digestion, the preparation was centrifuged at 300 g for 1 min (brake 3). The supernatant, containing the small cells, was filtered (40 μM) and the pellet discarded. The cell suspension was then topped up to 30 ml with incubation buffer (basic buffer (as above), 0.5% bovine serum albumin (BSA), pH 7.3) and spun at 1500 rpm for 7 min (brake 7) after which the supernatant was discarded and the pellet re-suspended in 1 ml incubation medium (PBS, 0.5% BSA, 2 μM EDTA). The small cell population was then sorted for the PIC cell population using magnetic-activated cell sorting (MACS) as per standard protocol (Miltenyi). Briefly, using direct mouse CD45 microbeads (Miltenyi) the CD45^+^ cells were depleted from the cell preparation, leaving the CD45^–^ fraction; from this, the Sca-1^+^ cells were then enriched using an indirect (FITC) mouse Sca-1 microbead kit (Miltenyi).

### Cell culture

Cells were cultured on 1.5% gelatin coated dishes in growth medium (45% Dulbecco’s modified Eagle’s medium (DMEM)/F12 Ham, 1× insulin-transferrin-selenium (ITS), 45% neurobasal medium, 0.5% glutamax, 1× B27, 1× N2, 20 ng/ml epidermal growth factor (EGF), 10 ng/ml basic fibroblast growth factor (bFGF), 10% embryonic stem cell qualified fetal bovine serum (ESCQ-FBS), 10 ng/ml leukaemia inhibitory factor (LIF), 1% penicillin/streptomycin, 0.1% fungizone, 0.1% gentamicin). Cultures were incubated at 37 °C in 5% CO_2_ and passaged 1 in 3 when they reached ~80% confluency using 0.25% trypsin-EDTA solution.

Mouse ESCs (gifted from Agi Grigoriadis, Kings College London) were cultured on 1% gelatin-coated dishes in ESC growth medium (10% FBS, 1 mM sodium pyruvate, MEM non-essential amino acids, 50 mM β-mercaptoethanol, 1000 U/ml GSK-inhibitor, 1000 U/ml MEK-inhibitor, 1000 U/ml LIF).

Single cell-derived clonal colonies were generated by serial dilution seeding of one cell per well of a 1.5% gelatin-coated 96-well tissue culture plate. Clonogenicity was assessed as a percentage of seeded wells that went on to form colonies.

### Directed differentiation

C9 PICS (P5) were plated at 2 × 10^4^ cells/cm^2^ in 10-cm^2^ dishes or four-well glass chamber slides as described below. Media were changed every 3 days.

Myogenic: 1.5% gelatin-coated dishes for 5 days in myogenic media; high-glucose (4.5 g/L) DMEM, 2% horse serum, 1% penicillin/streptomycin.

Endothelial: 10 μg/ml fibronectin-coated dishes for 7 days in endothelial media; DMEM, 10 ng/ml vascular endothelial growth factor (VEGF), 1% penicillin/streptomycin.

Hepatic: 10 μg/ml fibronectin-coated dishes for 14 days in hepatic media; low-glucose DMEM, 25% F12K media, 20 ng/ml hepatocyte growth factor (HGF), 10 ng/ml oncostatin, 1× ITS, 5 mM nicotinamide, 2.5% FBS, 1% penicillin/streptomycin.

Neuronal: 1 μg/ml laminin-coated dishes for 14 days in neuronal medium: low-glucose DMEM, 10% horse serum, 300 ng/ml retinoic acid.

### GFP transduction

C9 PICs (P10) were transduced with the construct for GFP via lentiviral transduction; 10^6^ 293 T cells at 70% confluency were treated with 5 μg Delta 8.9 plasmid, 10 μg GFP plasmid, 2 μg VSV-g plasmid (pre-prepared plasmids gifted from Daniele Torella, Magna Graecia University, Italy), and 30 μl Lipofectamine2000 in 6 ml of OptiMEM-I for 4 h. A further 5 ml of OptiMEM-I containing 10% FBS and 1% penicillin/streptomycin was then added. After 24 h the media was changed for normal 293 T growth media and left for a further 24 h, after which the lentiviral supernatant was collected. PICs were transduced for 24 h using a 1: 5 ratio of lentiviral supernatant:PIC media containing 8 μg/ml polybrene. The transduction efficiency was checked by flow cytometry.

### Tumourigenicity assay

Twelve-week-old C57BL/6 were anaesthetised via inhalation of isoflurane and immediately given antibiotics (0.5 ml Betamox LA) and pain relief (5 mg/kg Carprieve) subcutaneously and Viscotears used on the eyes to prevent drying. Animals were placed on a heat mat throughout surgery and recovery. Hair was removed prior to incision using clippers and Nair™ hair removal cream (3 min) before disinfection with Hibiscrub. A small (~10 mm) incision was made midline on the mouse right hand side just under the ribcage, followed by a slightly smaller incision (~7 mm) in the peritoneum directly underneath. The kidney was then gently exposed using small non-toothed forceps to hold it in place. A small incision was made to the kidney capsule using a 27 gauge needle without piercing the kidney itself. The kidney capsule was kept moist with PBS containing 0.05% gentamicin throughout. A small space was then made between the kidney and its capsule using a fine pipette tip containing the cell mix. The cell mix (1 × 10^6^ cells in 10 μl of 70% matrigel) was deposited slowly, forming a gel-like ball as the matrigel solidified, and the entry site cauterized to prevent leakage. The peritoneum and skin were individually closed using 5-0 Vicryl sutures and the animal allowed to recover. Ten out of 12 animals that were operated on survived (one did not recover, one died 24 h post-surgery). Animals were sacrificed 4 weeks post-surgery and assessed for tumour formation.

### Flow cytometry

Cells were blocked with 10% donkey serum in incubation medium (PBS, 0.5% BSA, 2 μM EDTA) immediately before incubation with the primary antibody. For nuclear expression of PW1, cells were permeabilised with BD fixation/permeabilisation kit as per the manufacturer’s instructions prior to the blocking step. Flow cytometry was conducted on a BD FACS Calibur and 10,000 events recorded for each analysis. Results were plotted and analysed using Flowing Software (Turku Centre for Biotechnology). See Additional file 2 (Table S2) for antibodies and controls used.

### Quantitative reverse-transcription polymerase chain reaction (qRT-PCR)

Total mRNA was obtained from cell pellets using the QIAshredders and RNeasy mini kit (Qiagen). Final mRNA concentration (ng/μl) and quality (260/280 and 260/230 ratio) of the resulting flow though was measured using a Nanodrop 2000.

cDNA was synthesised via reverse transcription, using a Taqman reverse transcription (RT) kit (Life technologies). qRT-PCR was performed on a Biorad i-cycler with a MyIQ detection system, using IQ SYBR Green supermix, 1 μl of template cDNA, and 500nM of forward and reverse primers with the following program:

1. 95 °C – 5 min
2. 40 cycles of:
  - 95 °C – 15 s
  - 60 °C – 30 s
  - 72 °C – 30 s

Florescence was detected as the end of each amplification cycle (step 2c).

Melt curve analysis was performed on all reactions at 0.5 °C increments between 55 and 95 °C to detect any genomic DNA contamination, primer dimers, and/or non-specific amplification. Data were analysed using BioRad IQ software, and the transcript copy number estimated by normalising results to the housekeeping gene (HKG) GAPDH, using the following equation where Ct = cycle threshold.

Copy number of target = 2500*1.93^(HKi – target Ct)

All primers were designed using NCBI Primer-BLAST software, with melting temperatures of 60 °C and primer lengths of ~20. All reactions were performed in triplicate. See Additional file 3 (Table S3) for primer sequences.

### Statistical analysis

Data are presented as mean ± SD. Significance between two groups was determined using an independent *t* test. For analysis of more than two groups, one-way analysis of variance (ANOVA) was performed with the Tukey post-hoc method to locate the differences. Significance was reported at *p* < 0.05.

## Results

### Abundance of PW1^+^ PICs and satellite cells decreases into old age

Expression of PW1^+^ PICs and satellite cells were assessed by immunohistochemistry on hind limb skeletal muscle cross-sections at 3, 10, and 21 days and 2 years. PW1^+^ cells were classified as being PICs located within the interstitial spaces, or satellite cells under the basal lamina, when counterstained with laminin (Additional file 4: Figure S1). To account for activated satellite cells within the interstitial spaces, Pax7 staining was employed; however no interstitial cells expressing both Pax7 and PW1 were found. A small fraction of interstitial PW1^+^/Pax7^–^ cells may be myoblasts in final stage mitosis.

At 3 days of age, ~9% of total nuclei were positive for PW1, with ~5% being PW1^+^ PICs and ~4% being PW1^+^ satellite cells. The abundance of PW1^+^ cells decreased with age to less than ~1.5% of total nuclei in 2-year-old animals. Most of these PW1^+^ cells (~1%) were identified as quiescent satellite cells (Additional file 4: Figure S1). Similarly, the number of PW1 nuclei per muscle fibre decreased with age from 1/3 fibres at 3 days to 1/31 fibres at 2 years. The decline in PW1^+^ PICs from 3 to 21 days was about 50%, going from ~5% of total nuclei at 3 days to 2.5% at 21 days (Additional file 4: Figure S1). In young mice between 3 and 21 days, PICs represented ~57% of all PW1^+^ cells, whilst this dropped significantly to ~30% in aged animals. In young mice between 3 and 21 days, satellite cells represented ~43% of PW1^+^ cells, but by 2 years this had increased to ~70% (Additional file 4: Figure S1). Therefore, the number of PW1^+^ PICs and PW1^+^ satellite cells decreases with age, yet the proportion of PW1^+^ quiescent satellite cells to PW1^+^ PICs increases, which suggests PW1^+^ PICs are declining at a faster rate with age. This could be because PW1^+^ PICs are taking up position as satellite cells to compensate for the decrease in satellite cells with age [30].

### PW1^+^ PICs express stemness markers and can be purified and propagated in vitro

Murine PICs are enriched in the Sca-1-positive (Sca-1^+^) CD45-negative (CD45^–^) fraction of muscle-derived cells, whereas the satellite cells reside in the Sca-1-negative fraction [17]. To isolate a purified population of young adult PICs we enzymatically digested murine hind limb muscle obtained from both legs of 21-day-old mice and, through MACS technology (Miltenyi), sorted the muscle-derived cells for the CD45-negative followed by the Sca-1-positive cell fraction. Approximately 800,000 CD45^–^/Sca-1^+^ PICs were obtained per mouse.

**Fig. 1:**
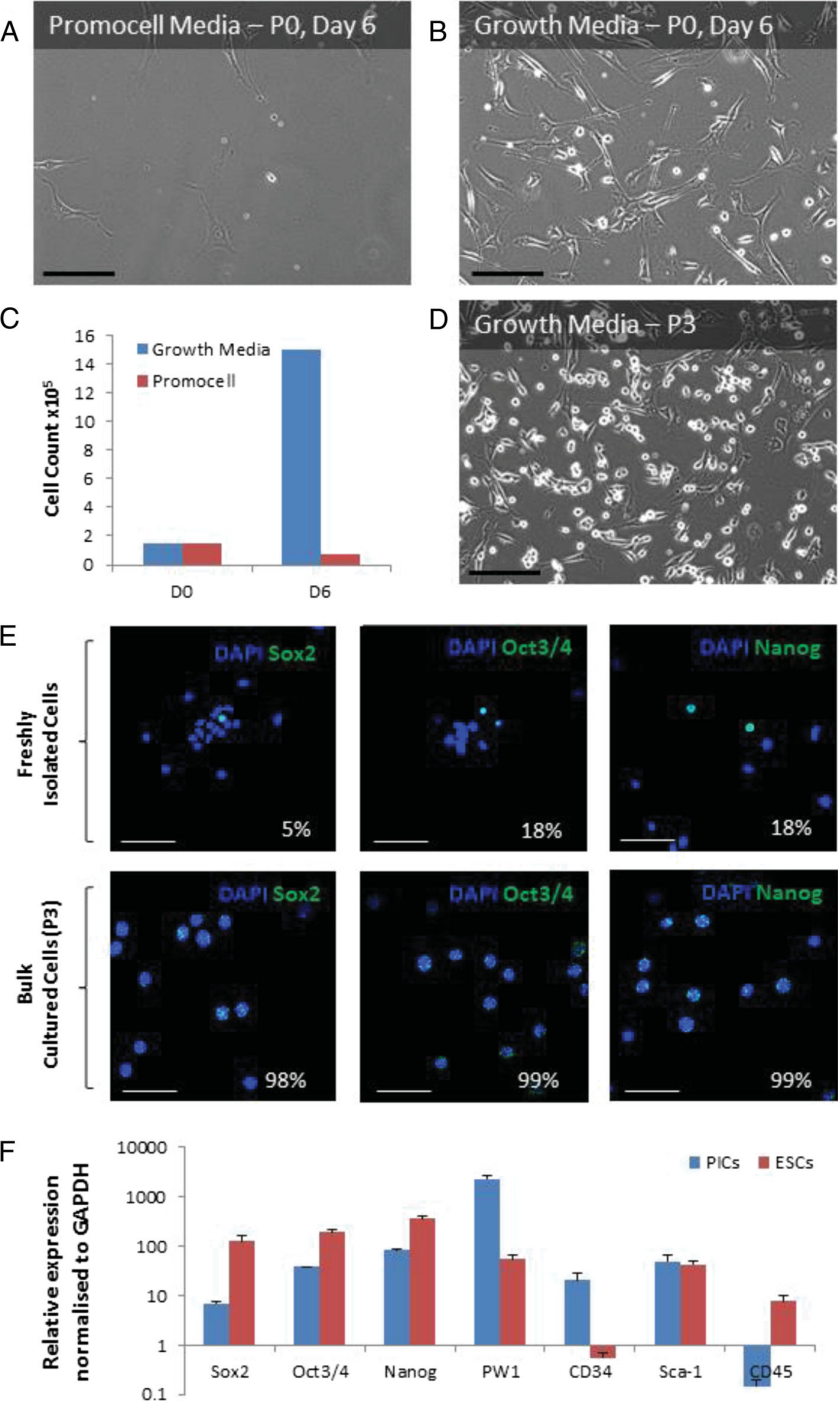
Growth and phenotype of CD45^–^/Sca-1^+^ PICs. **a**,**b** Representative light microscope images of CD45^–^/Sca-1^+^ positive interstitial cells (*PICs*) isolated from 21-day-old mice after 6 days in culture with Promocell media (**a**) or growth media (**b**). *Scale bars* = 200 μm. **c** Cell count at initial plating (*D0*) and after 6 days (*D6*) of culture in Promocell or PIC growth media (*n* = 1 culture dish). **d** Representative light microscope image of CD45^–^/Sca1^+^ PICs cultured to passage 3 (*P3*) in growth media. *Scale bar* = 200 μm. **e** Representative confocal micrograph of freshly isolated and cultured (P3) PICs showing expression of the pluripotency markers Sox2, Oct3/4, and Nanog. Quantification of positive cells is percentage of total cells counted. *Scale bar* = 50 μm. **f** qRT-PCR transcript analysis for PICs at P3 and mouse embryonic stem cells (*ESCs*). Bars represent the mean relative expression normalised to GAPDH. Error bars represent the standard deviation of the mean; *n* = 3

We tested two different cell media for the propagation of CD45^–^/Sca-1^+^ PICs; 15,000/cm^2^ CD45^–^/Sca-1^+^ PICs were plated in either complete skeletal muscle media purchased from Promocell or a defined stem/progenitor cell growth media, optimised and used by our laboratory [31] (Fig. 1a and b). After 6 days, cells cultured in Promocell media did not expand and showed a >50% decrease in number and appeared large and flattened (Fig. 1a and c). In contrast, CD45^–^/Sca-1^+^ PICs plated in our stem/progenitor cell growth media contained a number of small round bright cells (Fig. 1b) and had increased 10-fold in number to 150,000/cm^2^ (Fig. 1c). By passage 3, the number of small round bright cells with a very high nucleus to cytoplasmic ratio, which adhered to the culture dish, had visibly increased (Fig. 1d). This increase in small rounded bright cells was maintained for the duration of time in culture (20 passages) and these small cells detached quickly when trypsinised with very little cell death occurring at each passage. In contrast, the cells maintained in Promocell media arrested growth, did not show any small rounded bright cells, and did not get past P0; when trypsinised, the cells did not detach from the dish.

To determine whether our stem/progenitor cell growth media purifies for a stem/progenitor cell population we characterised CD45^–^/Sca-1^+^ PICs for the markers of stemness and pluripotency (Sox2, Oct3/4 and Nanog) at P0 (freshly isolated) and P3. Freshly isolated cells expressed Sox2 (5%), Oct3/4 (18%), and Nanog (18%); however, by P3 all (>98%) of the cells were positive for Sox-2, Oct3/4, and Nanog (Fig. 1e). In addition, gene transcription was assessed by qRT-PCR and compared to that of murine ESCs. As expected, CD45^–^/Sca-1^+^ PICs expressed transcripts for PW1, Sca-1, and CD34, whilst being negative for CD45 (transcript copy number < 1). CD45^–^/Sca-1^+^ PICs expressed transcripts for Sox2, Oct3/4, and Nanog, although all were lower than levels present in ESCs (Fig. 1f).

### PW1^+^ PICs maintain a PIC phenotype over culture time

**Fig. 2:**
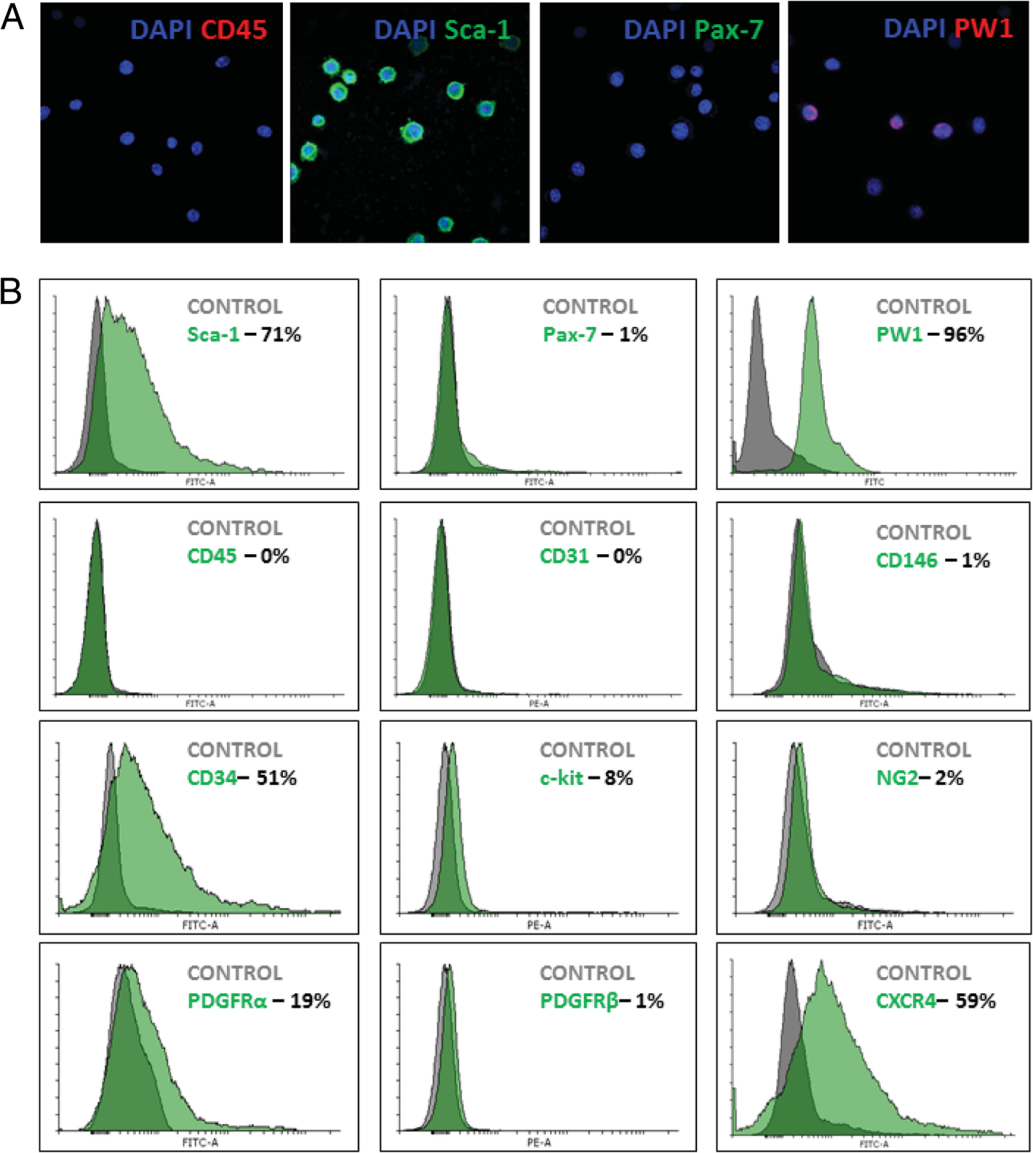
PICs retain their phenotype after three culture passages. **a** Representative confocal micrograph showing CD45^–^/Sca-1^+^ PICs at P3 are negative for CD45 and Pax-7 and positive for Sca-1 and PW1. *Scale bar* = 50 μm. **b** Flow cytometric analysis of CD45^–^/Sca-1^+^ PICs at P3 for the cell surface markers Sca-1, CD45, CD34, PDGFRα, CD31, c-kit, PDGFRβ, CD146, NG2, and CXCR4 and the nuclear markers Pax7 and PW1. *Grey* histogram represents the isotype control and the *green* histogram represents protein of interest in all histograms

To ensure that propagation in culture did not change the phenotype of CD45^–^/Sca-1^+^ PICs, we analysed CD45^–^/Sca-1^+^ PICs at P3 for an array of markers previously described in PIC populations using immunocytochemistry and flow cytometry analysis. In agreement with a PIC phenotype, CD45^–^/Sca-1^+^ cells at P3 were confirmed as being CD45^–^, Pax-7^–^, Sca-1^+^, and PW1^+^ (Fig. 2a). Flow cytometry confirmed these data, and also showed that PICs did not express the endothelial markers CD31 or CD146, nor the pericyte markers NG2 and PDGFRβ (≤2%), but did express PDGFRα (19%), CD34 (51%), and CXCR4 (59%) (Fig. 2b). A low percentage (8%) of CD45^–^/Sca-1^+^ cells also expressed the stem cell factor receptor, c-kit (Fig. 2b).

### PW1^+^ PICs are clonogenic and self-renewing

**Fig. 3:**
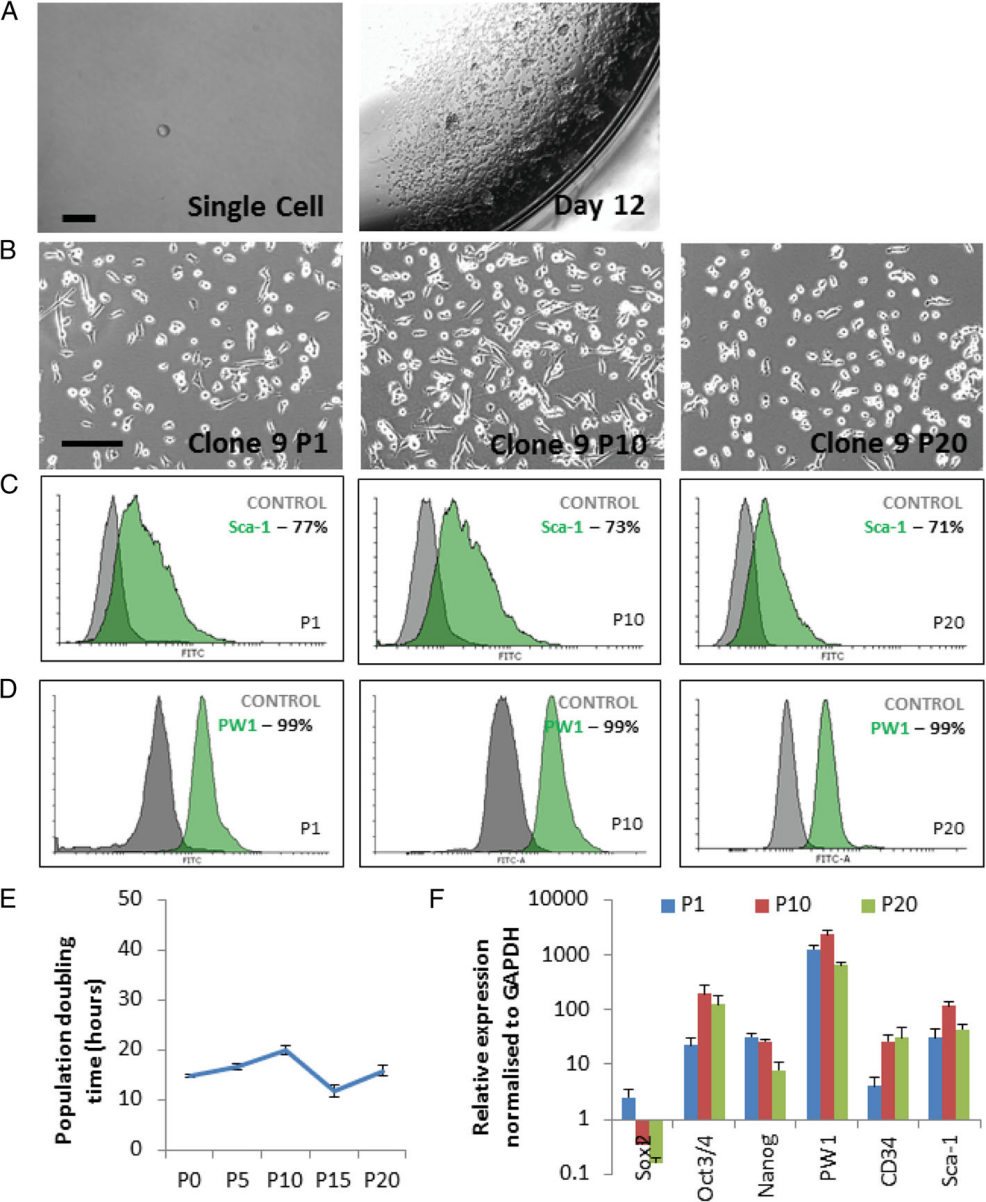
PICs are clonogenic and clones can be maintained over long-term culture. **a** Representative light microscope image of a single PIC in a well of a 96-well plate (*scale bar* = 100 μm), and subsequent clonal population after 12 days of culture (*scale bar* = 500 μm). **b** Representative light microscope images of C9 PIC morphology at passage (P)1, P10, and P20. *Scale bar* = 200 μm. **c**,**d** Flow cytometric analysis of C9 PICs for Sca-1 (**c**) and PW1 (**d**) at P2, P10, and P20. **e** Population doubling time of C9 PICs over 20 passages. Data are mean ± SD, *n* = 3. **f** qRT-PCR transcript analysis of C9 PICs at P1, P10, and P20. Bars represent the mean relative expression normalised to GAPDH. Error bars represent the standard deviation of the mean; *n* = 3

PICs that had been propagated to passage 3 were deposited as a single cell/well by serial dilution into 96-well cloning plates. After 12 days the number of colonies derived from each well was quantified; 34 ± 11% of single PICs formed clonal colony populations of small rounded cells, which formed aggregates at high density (Fig. 3a). Seven clones were picked for further analysis. The clones all expressed high levels of PW1 and Sca-1, maintaining a PIC phenotype (Additional file 5: Figure S2). The clones also expressed comparable transcript levels of PW1, Sca-1, CD34, Oct3/4, and Nanog compared to bulk cultured PICs when analysed by qRT-PCR (Additional file 6: Figure S3). However, only one clone (C9) maintained expression of Sox-2 (Additional file 6: Figure S3). C9 was selected for further characterisation and propagated for over 20 passages, maintaining its morphology (Fig. 3b), and expression of Sca-1 and PW1 at passages 1, 10, and 20 (Fig. 3c and d). Flow cytometry analysis for surface markers previously screened for in bulk PICs was conducted at P2 and P20 on C9. When compared to bulk PICs, C9 PICs had increased expression of CD34 (~6% increase) and CXCR4 (~20% increase), whilst C9 PICs no longer expressed PDGFRα (Additional file 7: Figure S4). There were no notable changes of more than 5% between P2 and P20 for any of the surface markers, showing that C9 maintained a stable phenotype over 20 passages (Additional file 7: Figure S4). Furthermore, C9 maintained a stable population doubling time of 16 ± 3 h over 20 passages, which equated to ~64 total population doublings over 20 passages (Fig. 3e). qRT-PCR analysis of C9 at P1, P10, and P20 showed a comparable transcript profile at each passage, with only the levels of Sox-2 markedly decreasing over culture time (Fig. 3f).

**Fig. 4:**
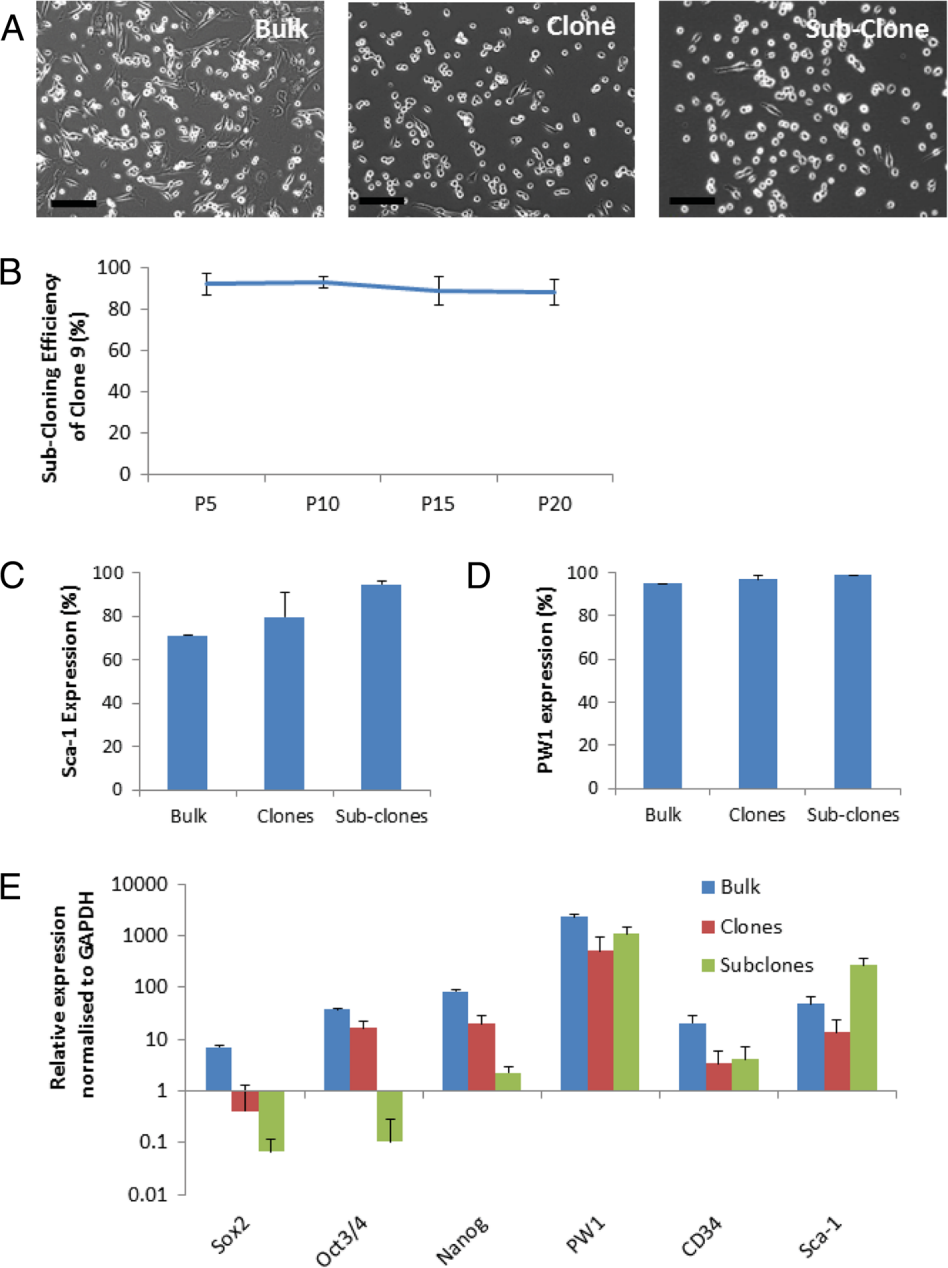
PIC sub-clone characterisation. **a** Representative light microscope images of bulk cultured, clonal, and sub-clonal PICs. **b** Sub-cloning efficiency of C9 PICs at every fifth passage up to passage (P)20. Data are mean ± SD; *n* = 3. Sca-1 (**c**) and PW1 (**d**) expression quantified by flow cytometry for bulk, clonal, and sub-clonal PICs. Data are mean ± SD; bulk, *n* = 1; clones, *n* = 7; sub-clones, *n* = 3. **e** qRT-PCR transcript analysis of bulk, clones, and sub-clones. Bars represent the mean relative expression normalised to GAPDH. Error bars represent the standard deviation of the mean; *n* = 3

C9 PICs deposited as a single cell in a 96-well plate produced single-cell derived sub-clones with an efficiency of 91 ± 2%, which was maintained when clonal efficiency was assessed at every fifth sub-cloning passage until passage 20 (Fig. 4a and b). PICs grown as bulk cells, their single-cell derived clones, and the single cell-derived sub-clones all maintained high levels of Sca-1 and PW1 expression when assessed by flow cytometry (Fig. 4c and d; Additional file 8: Figure S5 and Additional file 9: Figure S6).

qRT-PCR analysis revealed that, although the expression of PW1, CD34, and Sca-1 was consistent between bulk cells, clones, and sub-clones, the expression of pluripotent markers Oct3/4, Sox-2, and Nanog gradually decreased (Fig. 4e), emanating into the subclones of C9 being negative for Sox-2, Oct3/4 and markedly reduced for Nanog (Additional file 9: Figure S6). As the expression of Oct3/4, Sox-2, and Nanog was >98% in bulk cells (Fig. 1e), one would expect that a single cell-derived clone from bulk cells would be positive for pluripotent markers. Clones maintained positivity for Oct3/4, Sox-2, and Nanog, and yet the levels were decreased compared to bulk PICs (Fig. 4e; Additional file 9: Figure S6). Therefore, the expression levels of the pluripotent transcripts must have decreased in the clonal and sub-clonal population over culture time.

### PW1^+^ PICs and their clonal derivatives are bi-potent, differentiating into twitching myotubes and smooth muscle cells

**Fig. 5:**
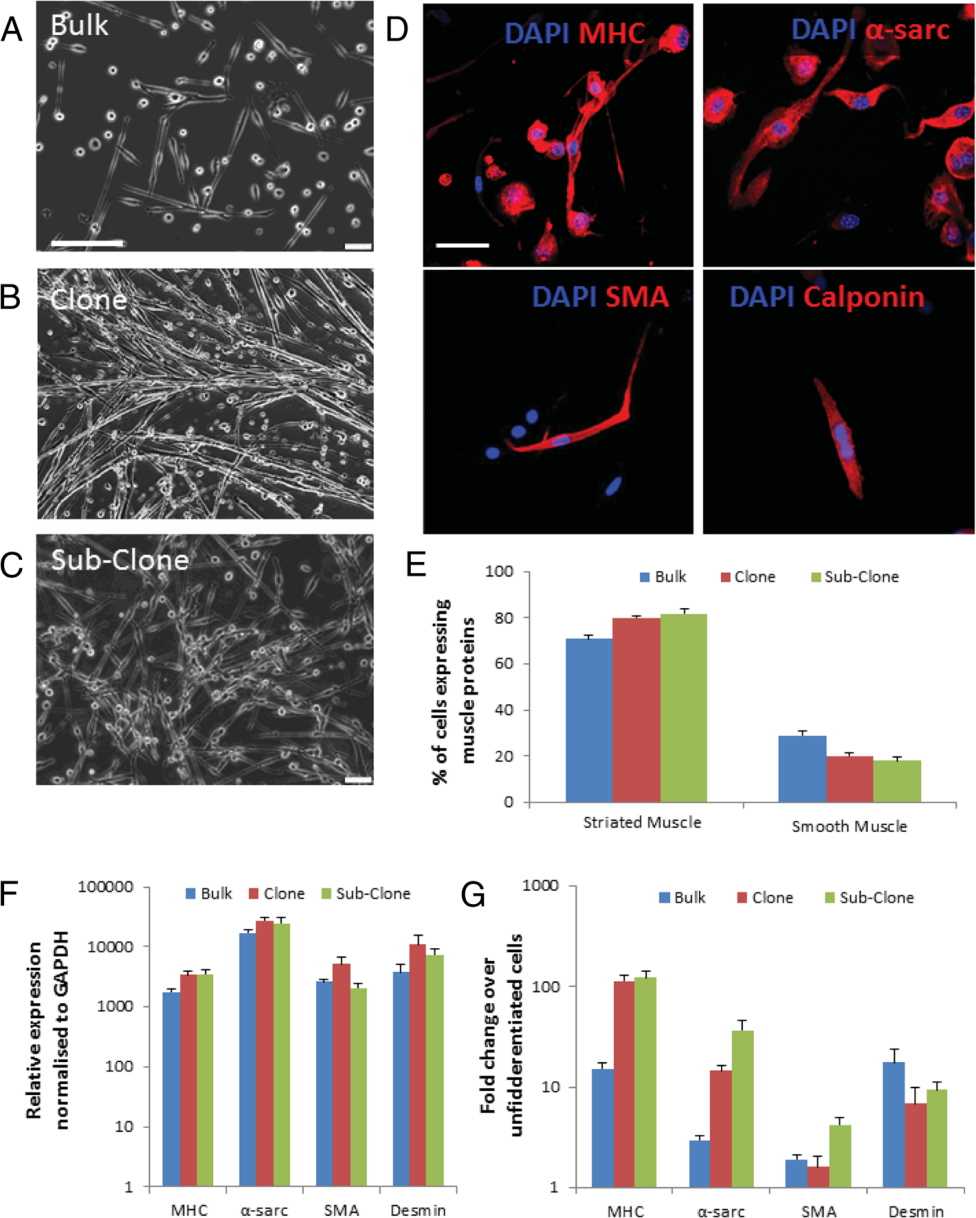
PICs are bi-potent. **a**–**c** Representative light microscope images of skeletal myogenic differentiated bulk (**a**), clonal (**b**), and sub-clonal (**c**) PICs. *Scale bar* = 200 μm. **d** Representative confocal micrograph showing differentiation of C9 PICs into skeletal muscle and smooth muscle lineage positive cells (myosin heavy chain (*MHC*), α-sarcomeric actin (*α-sarc*), smooth muscle actin (*SMA*), and calponin, all *red*). *Scale bar* = 50 μm. **e** Quantification of differentiated cells in bulk, clonal, and sub-clonal PIC populations. Data are mean ± SD; *n* = 3. (**f**) qRT-PCR transcript analysis for muscle markers MHC, α-sarc, SMA, and desmin after 5 days of directed myogenic differentiation of bulk, clonal, and sub-clonal PICs. Bars represent the mean relative expression normalised to GAPDH. Error bars represent the standard deviation of the mean; *n* = 3. **g** Fold-change in transcript copy number for MHC, α-sarc, SMA, and desmin over undifferentiated PICs. Data are mean ± SD; *n* = 3



When placed in myogenic differentiation media, bulk PICs, clonal PICs, and sub-clone PICs all formed myotubes, which were found to beat or twitch after 4 days of differentiation (Fig. 5a–c). However, efficiency of myotube formation and twitching was more pronounced in clonal and sub-clonal PICs than in bulk PICs (Fig. 5a–c; Additional file 10: Video). Differentiated cells were fixed and stained for MHC and α-sarcomeric actin to show striated muscle differentiation, and smooth muscle actin (SMA) and calponin to show smooth muscle differentiation of PICs (Fig. 5d). Bulk, clonal, and sub-clonal PICs showed increased differentiation into striated muscle (80% of cells expressed myosin heavy chain (MHC) and α-sarcomeric actin) compared to smooth muscle (~20% of cells expressed SMA and calponin) (Fig. 5e). Similar increases were also seen in the transcripts for striated and smooth muscle differentiation (Fig. 5f and g). These findings show that, in this case, single-cell derived cloning selects for a myogenic population of PICs.

**Additional file 10:** PW1 PICs form twitching myotubes after directed differentiation in vitro. Video: C9 cells after 5 days of directed myogenic differentiation. (AVI 61204 kb)


### PW1^+^ PICs have a broad developmental plasticity

PICs are bi-potent, differentiating into smooth muscle cells and skeletal muscle fibres, which are cell types that originate from the mesoderm layer. The ability of PICs to differentiate the trans-germ layer into cell types from the other two germ layers, the endoderm and ectoderm, has not yet been described. As PICs express the pluripotency markers Oct3/4, Nanog, and Sox2 (Fig. 1e and f) we sought to assess their trans-germ layer differentiation potential and potency in vitro and in vivo.

**Fig. 6:**
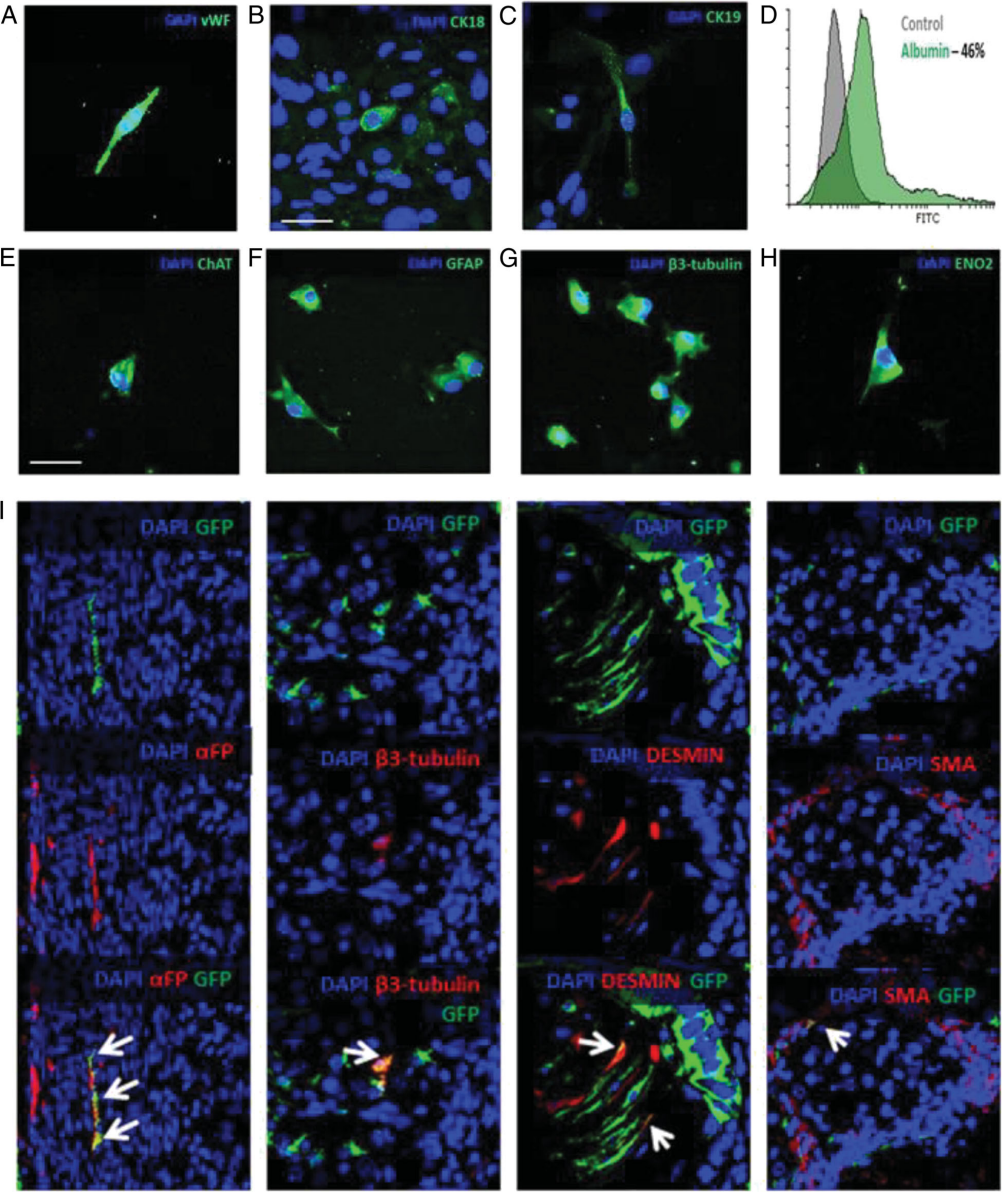
PICs show multi-potent potential in vitro and in vivo. **a**–**h** Representative confocal micrographs of C9 PICs following directed differentiation into endothelial (**a**), hepatic (**b**,**c**) and neuronal (**e**–**h**) lineages. **d** Flow cytometric analysis of C9 PICs for albumin expression after 14 days of hepatic differentiation. **i** Immunohistochemical staining of teratomas from PIC/ESC-treated mice showing GFP^+^ cells expressing markers of the three germ layers. *Arrows* indicate co-staining of green fluorescent protein (*GFP*) with αFP (endoderm), β3-tubulin (ectoderm), desmin, and smooth muscle actin (*SMA*) (mesoderm). *Scale bars* = 50 μm in all micrographs. *ChAT* choline acetyltransferase, *CK* cytokeratin, *ENO2* gamma-enolase, *GFAP* glial fibrillary acidic protein, *vWF* von Willebrand factor

First, we showed that PW1^+^ PICs differentiate into non-myogenic cells from the mesoderm layer, such as endothelial cells (Fig. 6a). To demonstrate cross-lineage differentiation into cell types of the endoderm lineage, C9 PICs were placed in hepatic differentiation media for 14 days, after which they displayed a change in morphology, and expressed proteins for cytokeratins 18 and 19 and albumin (Fig. 6b–d). Neurogenic differentiation was selected to determine the differentiation potential of PICs into cell types of the ectoderm lineage. After 14 days of directed neurogenic differentiation, a small number of cells became spindle shaped with dendritic projections as seen in neuronal cell types and expressed proteins for choline acetyltransferase (ChAT), glial fibrillary acidic protein (GFAP), class III beta tubulin (β-3-tubulin), and gamma-enolase (ENO2) (Fig. 6e–h). Differentiated PICs also showed increased transcript expression for each lineage compared to undifferentiated PICs (Additional file 11: Figure S7).

Next, we determined the trans-germ layer differentiation potential and potency of PICs in vivo utilising the teratoma assay where PICs were transplanted under the kidney capsule alongside ESCs. To distinguish between cells that arose from ESCs or PICs, C9 PICs (P10) were transduced with a GFP construct prior to injection. Post-transduction, GFP^+^ PICs demonstrated normal PIC morphology and their GFP expression was ~99%, confirmed at P2 post-transduction (Additional file 12: Figure S8). GFP^+^ PICs were propagated for 8 passages to obtain enough cells to perform the assay. Mouse ESCs were cultured feeder-free prior to transplantation to prevent MEF contamination.

Mice injected with PBS (no cells) or those injected with GFP^+^ PICs only did not form tumours, demonstrating that PICs are not tumorigenic (Additional file 12: Figure S8). In contrast, mice injected with ESCs, and those injected with a 50/50 mix of ESCs and GFP^+^ PICs, displayed hair loss and tumour formation, which contained a variety of cell types with different morphologies (Additional file 12: Figure S8). Indeed, mononuclear PIC derivatives, detected with anti-GFP antibody, were seen as mesodermal, ectodermal, and endodermal lineages (Fig. 6i), supporting a broad plasticity of PICs. However, the majority of GFP^+^ cells expressed mesodermal markers (desmin-positive, SMA-positive), reflecting PIC bi-potency in vivo (Fig. 6i).

## Discussion

The main findings that emanate from this study are: 1) PICs can be successfully isolated from hind limb muscle; 2) PICs can be propagated in culture for long periods whilst maintaining a stable phenotype; 3) PICs are clonogenic and self-renewing in vitro; 4) PICs express markers of pluripotency; 5) PICs display multipotent differentiation potential expressing proteins and transcripts from cells of the three germ layers in vitro and in vivo; 6) clonal PICs are primarily driven towards a mesodermal and specifically skeletal muscle lineage; and 7) PICs are not tumorigenic.

Although the number of PICs decreases with age (Additional file 4: Figure S1), there are still adequate numbers into young adulthood to efficiently isolate enough PICs for their propagation in a defined stem/progenitor cell growth culture media. This study is the first to show that murine PICs can be propagated and maintained in long-term culture. The media used is one optimised by our group and has previously been used to culture porcine PICs (pPICs) [18] and cardiac stem/progenitor cells [29, 31]. Indeed, we previously demonstrated pPICs were capable of maintaining a stable phenotype and karyotype over 40 passages [18]. The importance of using the correct growth media was further reinforced in this study by the growth senescence of PICs when placed in promocell growth media (Fig. 1).

Clonal PIC cell lines displayed a similar morphology and phenotype to both bulk PICs and each other, as did their sub-clonal counterparts. A clonal population was chosen for assessment over time in culture to ensure that results were characteristic of PICs and were not an artefact of a mixed cell population. C9 was subsequently chosen for further analysis due to it being the most similar to the transcription profile of non-cloned ‘bulk’ cells, and the phenotype of PICs in general. The ability to propagate PICs in vitro is in contrast to that seen in satellite cells which rapidly lose their self-renewal properties and undergo differentiation [32]. These data favour the use of PICs over satellite cells for expansion and their use in cell regenerative therapeutic settings.

When propagated in culture, PICs displayed characteristics of stem cells, being clonogenic and self-renewing. PICs demonstrated an ability to form single cell-derived clonal populations; their clonal efficiency of ~34% was less than reported in pPICS (53%) [18], but is within the range previously reported for other adult stem/progenitor cell populations, such as porcine skin stem cells [33]. Moreover, C9 PICs retained a subsequent clonogenicity of ~91%, substantiating their clonogenic properties, and self-renewal ability. This is the first study to propagate and compare clones, and subsequently sub-clone PICs.

C9 PICs exhibited a stable population doubling (PD) over 20 passages, demonstrating that each generation is as proliferative as its predecessor. A shorter PD is indicative of a higher rate of proliferation: the PD of murine ESCs is ~10–14 h [34], porcine iPSCs derived from fetal fibroblasts have a PD of ~17 h [35], whilst pPICS have a PD of ~22 h [18]. The doubling time of PICs isolated from the mouse (~16 h) was shorter than that reported in pPICs, which could be because murine PICs are more proliferative than pPICs or the pPIC population was not a clonal cell line and was not sorted for Sca-1, and was therefore heterogeneous. In heterogeneous populations, different cell types divide at different rates, therefore affecting the overall doubling time. In contrast, C9 PICs were derived from a single cell and thus all cells should display a similar rate of proliferation.

The total number of population doublings over 20 passages was calculated to be ~64, far surpassing Hayflick’s limit of 14–28 doublings for cells of murine origin, as well as also extending beyond the 40–60 doublings initially reported as the maximum in human cells [36]. Importantly, in addition to maintaining their PD, C9 PICs also retained their morphology and phenotype over time in culture, displaying comparable levels of PW1 and Sca-1 at P2 and P20.

This is the first study to describe the presence of pluripotency markers in murine PICs. As expected, the levels of pluripotency gene transcripts were lower in PICs than in ESCs, although they were still substantial. It was noted that there was a decrease in transcript levels of all three pluripotency genes over time in culture in clonal and sub-clonal PICs. Whether the pluripotent potential of the cells differed with time was not tested. Despite these discrepancies, the growth kinetics and morphology did not change. This therefore suggests they could be artefacts of the in vitro environment rather than an inability of the PICs to self-renew competently. In vivo PICs would naturally inhabit a three-dimensional environment interacting with many other cell types and in vitro conditions cannot currently provide an identical niche to that in which they naturally reside within the tissue of origin. Bulk PICs had the highest number of transcripts for pluripotency markers; however, bulk PICs were not cultured long term. Therefore, it is not known if PICs self-renew more efficiently in a heterogeneous culture.

This study demonstrates that a single PIC is capable of generating both types of muscle—striated and smooth. This may also account for the difference in ratio with the clonal cell line used displaying an affinity towards skeletal muscle, whilst the previously described heterogeneous populations harbour cells that form both types [17, 18, 20]. Aside from the differences in ratio, all studies are in agreement that PICs are capable of forming both skeletal and smooth muscle. Importantly, the observation of twitching fibres at 5 days of differentiation indicates functionality of the muscle fibres and not just phenotypic and biochemical expression.

In the present study, clonal PICs expressed low PDGFRα, whereas the PICs reported by Lewis et al. [18] and Pannérec et al. [20] expressed PDGFRα. The expression of PDGFRα could determine the differentiation capacity of PICs into either skeletal or smooth muscle [17, 20]. Indeed, Pannérec et al. [20] reported that PDGFRα-positive PICs were capable of adipogenic differentiation but did not display myogenic differentiation potential, whilst PDGFRα-negative PICs had myogenic potential but lacked adipogenic differentiation capability. Furthermore, these findings are in agreement with previous data that PDGFRα^+^ cells in adult muscle interstitium are fibro-adipogenic progenitor (FAP) cells [16, 37]. With regard to skeletal and smooth muscle differentiation, the present study is the first to show the direct endothelial cell differentiation potential of PICs. However, full differentiation and functional maturity was not tested.

For the first time, this study demonstrated that PICs can cross the germ layer barrier and contribute to the formation of cell types from all three lineages in vitro and in vivo. However, whilst these assays suggest a broad differentiation potential with upregulation of lineage specific markers for all three germ layers, they do not demonstrate any functionality of PIC-derived differentiated cells. In both differentiation assays PICs showed a greater affinity towards the mesodermal lineage, with higher transcript levels and fold changes in vitro, and more PICs co-expressing mesodermal proteins, than endodermal or ectodermal ones in vivo. Furthermore, in vitro differentiation resulted in immature cells suggesting that either PICs are not capable of full differentiation due to intrinsic processes, or more efficient differentiation conditions (i.e. three-dimensional environment, scaffolds) are needed to drive full maturation.

The ability to cross the germ-layer barrier is rare but not unknown in adult stem cell populations. In 2007, Beltrami et al. demonstrated that Oct3/4^+^/Nanog^+^/REX1^+^ human multipotent adult stem cells (hMASCs), isolated from the liver, heart, and bone marrow, were capable of cross-lineage differentiation in vitro, each demonstrating differentiation into neuron (ectoderm)-, osteoblast (mesoderm)-, and hepatocyte (endoderm)-like cells [38]. Similarly, murine PDGFRα^+^ cardiac resident cCFU cells are capable of hepatic, endoderm, and neuronal differentiation in vitro. Furthermore, when GFP-labelled cCFUs cells were transplanted alongside unlabelled ESCs under the kidney capsule of a mouse, resulting teratomas contained GFP-expressing cells from all three germ layer lineages [39]. Moreover, muscle-derived stem cells, obtained via pre-plating, have previously been shown to differentiate into hepatocyte-like cells when co-cultured with hepatocytes, expressing albumin and HNF1α [40]. When injected into liver tissue following partial hepatectomy, they engraft and persist >3 months post-transplantation [40]. The functionality of cells generated in vitro is hard to determine, and behaviour in vitro does not necessarily encapsulate the behaviour of their in vivo counterparts. Importantly, Dil-labelled adult neural stem cells have shown transdermal differentiation, contributing to the formation of chimeric chick and mouse embryos, and to cell formation of all three germ layers [41]. This suggests that some adult stem cell populations are capable of generating functional cells of multiple lineages.

PICs did not form teratomas in vivo, suggesting they have multipotent potential, but are not pluripotent, as also seen in PDGFRα^+^ cardiac cCFUs cells [39]. Importantly, the inability of PICs to form teratomas independently means they are good cell candidates for cellular therapies. It is yet to be tested whether their pluripotent capability would have any unwanted consequences in tissue-specific regeneration, however previous studies using PICs to regenerate damaged skeletal muscle in both mice and pigs have not reported any [17, 18].

Whilst PICs have previously been shown to contribute to the formation of new smooth and skeletal muscle upon acute injury [17, 18], it has not yet been determined if they are suitable and effective for treating chronic conditions such as muscular dystrophy. Therefore, the next step is to elucidate the therapeutic potential of PICs using the mdx mouse model. Furthermore, the bi-potent differentiation potential of PICs makes them an ideal cell candidate to engineer skeletal muscle tissue constructs in vitro. A difficulty of generating skeletal muscle tissue constructs in vitro is the formation of perfusable blood vessels within the tissue construct. PICs, when stimulated under the right cues, would be able to differentiate into either smooth muscle or striated skeletal muscle. It has been shown that co-culture of C2C12 myoblasts, embryonic fibroblasts, and endothelial cells on highly porous and biodegradable polymer scaffolds resulted in the formation of endothelial networks within engineered muscle tissues in vitro and enhanced vascularization, blood perfusion, and survival of the tissue constructs after implantation [42]. Electrical stimulation and force production are also important cues for skeletal muscle development and maturation. Therefore, the ability of PICs (in co-culture with endothelial cells) to engineer skeletal muscle in vitro should be tested using biocompatible hydrogels/scaffolds together with the use of electrical pulse stimulation [43] to recapitulate the in vivo niche.

## Conclusion

In conclusion, murine PICs display properties of bona fide tissue-specific stem/progenitor cells, being clonogenic, self-renewing, and multipotent in vitro and in vivo. However, clonal PICs display a strong preference towards the skeletal muscle lineage from which they originate. Furthermore, PICs are not tumorigenic. These data validate the ability of isolated PICs to undergo in vitro propagation with the prospect of generating large numbers of these cells to be used in cellular regenerative therapies to treat a variety of diseases.

## Additional files

Additional file 1: Table S1. List of all antibodies used in immunohistochemistry and immunocytochemistry. (PDF 90 kb)

Additional file 2: Table S2. List of all antibodies and controls used in flow cytometry. (PDF 93 kb)

Additional file 3: Table S3. List of primers used. (PDF 89 kb)

Additional file 4: Figure S1. Identification and quantification of PICs and satellite cells in murine hind limb muscle. (A–C) Immunohistochemistry of paraffin-embedded hind limb muscle from 10-day-old mice identifies PW1^+^ cells. PICs are located within interstitial spaces (B) and satellite cells located under the basal lamina (C). (D,E) Quantification of PW1^+^ PICs and satellite cells in the hind limb muscle of mice at 3, 10, and 21 days, and 2 years of age, expressed as a percentage of total nuclei (D) and per 100 muscle fibres (E). (F) Ratio of PICs to satellite cells in neonatal to aged mice. Data are mean ± SD; *n* = 3 per group. (PDF 262 kb)

Additional file 5: Figure S2. Characterisation of clonal PICs. (A) Morphology of 7 PIC clones observed using light microscopy. Scale bar = 200 μm. (B) Flow cytometric analysis of PW1 expression in the seven clonal PIC populations. (C) Flow cytometric analysis of Sca-1 expression in the seven clonal PIC populations. (PDF 140 kb)

Additional file 6: Figure S3. Transcript analysis of clonal vs. bulk PICs. (A) qRT-PCR transcript analysis of PIC markers PW1, CD34, and Sca-1 in clonal PICs, compared to bulk PICs. (B) qRT-PCR transcript analysis of pluripotency markers Oct3/4, Sox2, and Nanog in clonal PICs compared to bulk PICs. Bars represent the mean transcript copy number normalised to GAPDH. Error bars represent the standard deviation of the mean; *n* = 3. (PDF 84 kb)

Additional file 7: Figure S4. Phenotypic stability of C9 over 20 culture passages. Flow cytometric analysis at (A) P2 and (B) P20 quantifying expression of CD34, PDGFRα, CXCR4, PDGFRβ, c-kit, CD31, CD146, and NG2 in C9 PICs. (PDF 168 kb)

Additional file 8: Figure S5. PW1/Sca-1 flow cytometric analysis of bulk, clonal, and sub-clonal PICs. (A) Bulk expression of PW1 and Sca-1 at P3. (B) C9 clone expression of PW1 and Sca-1 at P2. (C) C9A sub-clone expression of PW1 and Sca-1 at P2. Plots are representative of: bulk, *n* = 1; clones, *n* = 7; sub-clones, *n* = 3. (PDF 145 kb)

Additional file 9: Figure S6. Flow cytometry and transcript analysis of C9 sub-clones vs. C9 PICs. (A) qRT-PCR transcript analysis of sub-clones (C9A–C) compared to C9 PICs. Bars represent the mean relative expression normalised to GAPDH. Error bars represent the standard deviation of the mean; *n* = 3. (B) Flow cytometry histograms show consistent expression of Sca-1 and PW1 in sub-clones (C9A–C). (PDF 124 kb)

Additional file 11: Figure S7. Transcript analysis of PICs differentiated into the three germ layers in vitro. (A–C) qRT-PCR analysis of the fold change increase in endothelial (A), hepatic (B), and neuronal (C) transcripts of differentiated C9 PICs, compared to undifferentiated PICs. (D) qRT-PCR analysis of myogenic, cardiomyogenic, endothelial, hepatic, and neuronal transcripts of undifferentiated C9 PICs. Bars represent the mean relative expression normalised to GAPDH. Error bars represent the standard deviation of the mean; *n* = 3. (PDF 82 kb)

Additional file 12: Figure S8. Multipotency of PICs in vivo. (A) Fluorescent microscope images of GFP-transduced C9 PICs. Scale bar = 200 μm. (B) Flow cytometric analysis of GFP expression in transduced C9 PICs (green histogram), compared to mock transduced cells (grey histogram). (C) Teratomas viewed on the kidney of mice after 4 weeks. No teratomas evident in SHAM (*n* = 2) or PIC-treated animals (*n* = 3). Teratoma formation observed in PIC/ESC (*n* = 3) and ESC-treated (*n* = 2) animals. (D) Variety of cell morphologies seen in teratomas, visualised by haematoxylin and eosin staining. Scale bar = 200 μm (top left) and 20 μm all other images. (E,F) GFP was detected in teratomas generated by GFP^+^ PIC/ESC-treated mice only and not in ESC-treated mice, shown by DAB staining (E) and immunofluorescence staining (F). Scale bar = 100 μm. (PDF 713 kb)

## Abbreviations

BSA: Bovine serum albumin
DMD: Duchenne’s muscular dystrophy
DMEM: Dulbecco’s modified Eagle’s medium
ESC: Embryonic stem cell
FBS: Fetal bovine serum
GFP: Green fluorescent protein
ITS: Insulin-transferrin-selenium
LIF: Leukaemia inhibitory factor
MACS: Magnetic-activated cell sorting
MHC: Myosin heavy chain
PBS: Phosphate-buffered saline
PD: Population doubling
PIC: PW1-positive interstitial cell
qRT-PCR: Quantitative reverse-transcription polymerase chain reaction
Sca: Stem cell antigen
SMA: Smooth muscle actin
