# Supplementary material for: Skeletal muscle-derived interstitial progenitor cells (PICs) display stem cell properties, being clonogenic, self-renewing, and multi-potent in vitro and in vivo
Source: Stem Cell Res Ther. 2017 Jul 4;8:158. doi: 10.1186/s13287-017-0612-4 (PMC5496597; doi:10.1186/s13287-017-0612-4)
Supplement: Supplementary file 2 — List of all antibodies used in immunohistochemistry and immunocytochemistry. (PDF 90 kb) [file 13287_2017_612_MOESM1_ESM.pdf]

Supplementary Table 1: List of all antibodies used in Immunohistochemistry and Immunocytochemistry.

Primary Antibody	Dilution	Incubation	Secondary Antibody
PW1 (Gifted by David Sassoon, Inserm, Paris)	1:3000	Overnight at 4°C	Cy 3 Donkey anti-Rabbit (Stratech)
Laminin (Sigma)	1:50	1 Hour at 37°C	Alexa Fluor 594 Donkey anti-Chicken (Stratech)
Sca-1 (Abcam)	1/50	1 hour at 37°	Alexa Fluor 488 Donkey anti-Rat (Stratech)
Sox2 (Santa Cruz)	1/50	1 hour at 37°	Dylight 488 Donkey anti-Goat (Stratech)
Nanog (Abcam)	1/50	1 hour at 37°	Alexa Fluor 488 Donkey anti-Rabbit (Stratech)
Oct3/4 (Santa Cruz)	1/50	1 hour at 37°	FITC Donkey anti-Mouse IgG (Stratech)
Pax7 (DSHB)	1/50	1 hour at 37°	FITC Donkey anti-Mouse IgG (Stratech)
CD45 (Santa Cruz)	1/50	1 hour at 37°	Alexa Fluor 594 Donkey anti-Rat (Stratech)
α-sarc (Sigma)	1/50	1 hour at 37°	Dylight 594 Donkey anti-Mouse IgM (Stratech)
MHC (Sigma)	1/50	1 hour at 37°	Cy3 Donkey anti-Mouse IgG (Stratech)
SMA (Sigma)	1/500	1 hour at 37°	Cy3 Donkey anti-Mouse IgG (Stratech)
Calponon (Sigma)	1/50	1 hour at 37°	Cy 3 Donkey anti-Rabbit (Stratech)
Desmin (Santa Cruz)	1/50	1 hour at 37°	Cy3 Donkey anti-Mouse IgG (Stratech)
vWF Millipore	1/50	1 hour at 37°	Alexa Fluor 488 Donkey anti-Rabbit (Stratech)
CK18 Abcam	1/50	1 hour at 37°	FITC Donkey anti-Mouse IgG (Stratech)
CK19 Santa Cruz	1/50	1 hour at 37°	Dylight 488 Donkey anti-Goat (Stratech)
ChAT Abcam	1/50	1 hour at 37°	Alexa Fluor 488 Donkey anti-Rabbit (Stratech)
GFAP Dako	1/50	1 hour at 37°	Alexa Fluor 488 Donkey anti-Rabbit (Stratech)
β3-Tubulin Abcam	1/50	1 hour at 37°	FITC Donkey anti-Mouse IgG (Stratech) or Cy3 Donkey anti-Mouse IgG (Stratech)
γ-Enolase Santa Cruz	1/50	1 hour at 37°	Dylight 488 Donkey anti-Goat (Stratech)
GFP (Abcam)	1/50	1 hour at 37°	Alexa Fluor 488 Donkey anti-Rabbit or HRP Donkey anti-Rabbit (Santa Cruz) (Stratech)
αFP (Life Technologies)	1/50	1 hour at 37°	Cy3 Donkey anti-Mouse IgG (Stratech)
