# Supplementary material for: Skeletal muscle-derived interstitial progenitor cells (PICs) display stem cell properties, being clonogenic, self-renewing, and multi-potent in vitro and in vivo
Source: Stem Cell Res Ther. 2017 Jul 4;8:158. doi: 10.1186/s13287-017-0612-4 (PMC5496597; doi:10.1186/s13287-017-0612-4)
Supplement: Supplementary file 3 — List of all antibodies and controls used in flow cytometry. (PDF 93 kb) [file 13287_2017_612_MOESM2_ESM.pdf]

Supplementary Table 2: List of all antibodies and controls used in flow cytometry.

Antibody	Conjugate/ Secondary	Control	Dilution	Incubation
PW1 Inserm, Paris	Alexa Fluor 488 Donkey anti-Rabbit Stratech	Alexa Fluor 488 Donkey anti-Rabbit Stratech	1/20	15 minutes at 4°C
Sca-1 Miltenyi	FITC conjugated	FITC Mouse IgG Isotype control Miltenyi	1/20	15 minutes at 4°C
Albumin Abcam	FITC Conjugated	FITC Rat IgG Isotype control Abcam	1/20	15 minutes at 4°C
CD45 Biolegend	FITC Conjugated	FITC Mouse IgG Isotype control Abcam	1/20	15 minutes at 4°C
Pdgfrβ Abcam	PE Conjugated	PE Mouse IgG Isotype Control Abcam	1/20	15 minutes at 4°C
CXCR4 Abcam	Alexa Fluor 488 Donkey anti-Rat Stratech	Alexa Fluor 488 Donkey anti-Rat Stratech	1/20	15 minutes at 4°C
NG2 Santa Cruz	Alexa Fluor 488 anti-Rabbit Stratech	Alexa Fluor 488 Donkey anti-Rabbit Stratech	1/20	15 minutes at 4°C
Pdgfrα Santa Cruz	Dylight 488 Donkey anti-Goat Stratech	Dylight 488 Donkey anti-Goat Stratech	1/20	15 minutes at 4°C
CD146 RnD	FITC Donkey anti-Mouse IgG Stratech	FITC Donkey anti-Mouse IgG Stratech	1/20	15 minutes at 4°C
c-kit Santa Cruz	Alexa Fluor 488 Donkey anti-Rabbit Stratech	Alexa Fluor 488 Donkey anti-Rabbit Stratech	1/20	15 minutes at 4°C
CD34 eBioscience	FITC Conjugated	FITC Rat IgG isotype Control Abcam	1/20	15 minutes at 4°C
CD31 Miltenyi	PE Conjugated	PE Rat IgG Isotype Control Miltenyi	1/20	15 minutes at 4°C
CD31 eBioscience	FITC Conjugated	FITC Rat IgG isotype Control Abcam	1/20	15 minutes at 4°C
Pax7 DSHB	FITC Donkey anti-Mouse IgG Stratech	FITC Donkey anti-Mouse IgG Stratech	1/20	15 minutes at 4°C
c-kit Miltenyi	PE Conjugated	PE Rat IgG isotype Control Miltenyi	1/20	15 minutes at 4°C
