# Supplementary material for: Skeletal muscle-derived interstitial progenitor cells (PICs) display stem cell properties, being clonogenic, self-renewing, and multi-potent in vitro and in vivo
Source: Stem Cell Res Ther. 2017 Jul 4;8:158. doi: 10.1186/s13287-017-0612-4 (PMC5496597; doi:10.1186/s13287-017-0612-4)
Supplement: Supplementary file 4 — List of primers used. (PDF 89 kb) [file 13287_2017_612_MOESM3_ESM.pdf]

Supplementary Table 3: List of primers used.

Gene	Accession Number	Forward	Reverse
Sox2	NM_011443.3	CACAACTCGGAGATCAGCAA	CTCCGGGAAGCGTGTACTTA
Oct3/4	NM_013633.2	CCAATCAGCTTGGGCTAGAG	CTGGGAAAGGTGTCCCTGTA
Nanog	NM_028016.2	TACCTCAGCCTCCAGCAGAT	GTGCTGAGCCCTTCTGAATC
PW1	NM_008817.2	TTTTGGTGAGTTGCTTGCAG	ACGTTCTTGGGCATAACTGG
CD34	NM_133654.3	GGGTAGCTCTCTGCCTGATG	TCTCTGAGATGGCTGGTGTG
SCA1	NM_001271446.1	CCATCAATTACCTGCCCCTA	AAGGTCTGCAGGAGGACTGA
CD45	NM_011210.3	CCTGCTCCTCAAACTTCGAC	GACACCTCTGTCGCCTTAGC
GAPDH	NM_008084.2	ACCCAGAAGACTGTGGATGG	CACATTGGGGGTAGGAACAC
β-actin	NM_007393.3	AGCCATGTACGTAGCCATCC	TCTCAGCTGTGGTGGTGAAG
α-sarc	NM_009606.2|	AAGTGCGACATCGACATCAG	AAGTGCGACATCGACATCAG
MHC	NM_001039545.2	CCAAGCTGACCAAGGAGAAG	CCAAGCTGACCAAGGAGAAG
SMA	NM_007392.3	GCTGTCCCTCTATGCCTCTG	GAAGGAATAGCCACGCTCAG
Desmin	NM_010043.1	TACACCTGCGAGATTGATGC	ACATCCAAGGCCATCTTCAC
CD31	NM_001032378.1	GCCTCACCAAGAGAACGGAAGGC	TGGGCCTTCGGCATGGAACG
vWF	NM_011708.4	TGCCCTTGTGTGTGCACGGG	GTACCCTGGCTGCTGCACCG
CD146	NM_023061.2	GAGCTCATCTCCCCTCACAG	TCCTGACCACTACCCAAAGG
Ck18	NM_010664.2	CGAGGCACTCAAGGAAGAAC	CTTGGTGGTGACAACTGTGG
Ck19	NM_008471.2	CTCGGATTGAGGAGCTGAAC	TCACGCTCTGGATCTGTGAC
Albumin	NM_009654.3	GACAAGGAAAGCTGCCTGAC	TTCTGCAAAGTCAGCATTGG
HNF1α	NM_009327.3	ACTTGCAGCAGCACAACAT	GAATTGCTGAGCCACCTCTC
β-3-tubulin	NM_023279.2	CATGGACAGTGTTCGGTCTG	TGCAGGCAGTCACAATTCTC
ChAt	NM_009891.2	GTAACAGCCCAGGAGAGCAG	AGGTGTTGCATGCACTGAAG
ENO2	NM_013509.2	TCTATCGCCACATTGCTCAG	AGGGTGTGGTACACCTCTGC
GFAP	NM_001131020.1	CACGAACGAGTCCCTAGAGC	ATGGTGATGCGGTTTTCTTC
