# Supplementary material for: Skeletal muscle-derived interstitial progenitor cells (PICs) display stem cell properties, being clonogenic, self-renewing, and multi-potent in vitro and in vivo
Source: Stem Cell Res Ther. 2017 Jul 4;8:158. doi: 10.1186/s13287-017-0612-4 (PMC5496597; doi:10.1186/s13287-017-0612-4)
Supplement: Supplementary file 5 — Identification and quantification of PICs and satellite cells in murine hind limb muscle. (A–C) Immunohistochemistry of paraffin-embedded hind limb muscle from 10-day-old mice identifies PW1+ cells. PICs are located within interstitial spaces (B) and satellite cells located under the basal lamina (C). (D,E) Quantification of PW1+ PICs and satellite cells in the hind limb muscle of mice at 3, 10, and 21 days, and 2 years of age, expressed as a percentage of total nuclei (D) and per 100 muscle fibres (E). (F) Ratio of PICs to satellite cells in neonatal to aged mice. Data are mean ± SD; n = 3 per group. (PDF 262 kb) [file 13287_2017_612_MOESM4_ESM.pdf]

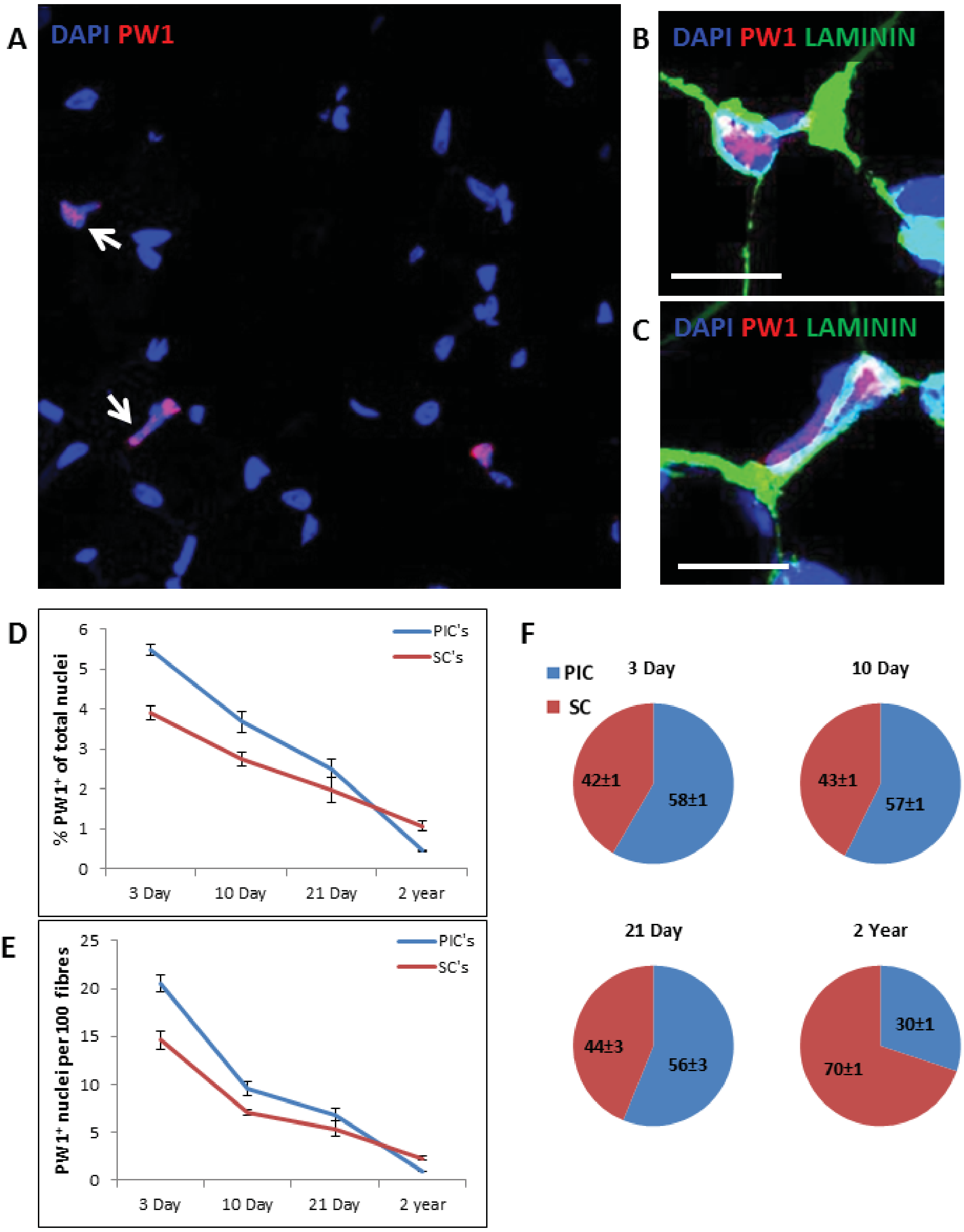

Supplementary Figure 1. Identification and quantification of PICs and satellite cells in murine hind-limb muscle. (**A-C**) Immunohistochemistry of paraffin embedded hind-limb muscle from 10 day old mice identifies PW1^{pos} cells. PICs are located within interstitial spaces (B) and satellite cells located under the basal lamina (**C**). (**D-E**) Quantification of PW1^{pos} PICs and satellite cells in the hind-limb muscle of mice at 3d, 10d, 21d and 2 years of age, expressed as a percentage of total nuclei (**D**) and per 100 muscle fibres (**E**). (**F**) Ratio of PICs to satellite cells in neonatal to aged mice. Data are Mean ± SD, n=3 per group**.**
