# Supplementary material for: Skeletal muscle-derived interstitial progenitor cells (PICs) display stem cell properties, being clonogenic, self-renewing, and multi-potent in vitro and in vivo
Source: Stem Cell Res Ther. 2017 Jul 4;8:158. doi: 10.1186/s13287-017-0612-4 (PMC5496597; doi:10.1186/s13287-017-0612-4)
Supplement: Supplementary file 6 — Characterisation of clonal PICs. (A) Morphology of 7 PIC clones observed using light microscopy. Scale bar = 200 μm. (B) Flow cytometric analysis of PW1 expression in the seven clonal PIC populations. (C) Flow cytometric analysis of Sca-1 expression in the seven clonal PIC populations. (PDF 140 kb) [file 13287_2017_612_MOESM5_ESM.pdf]

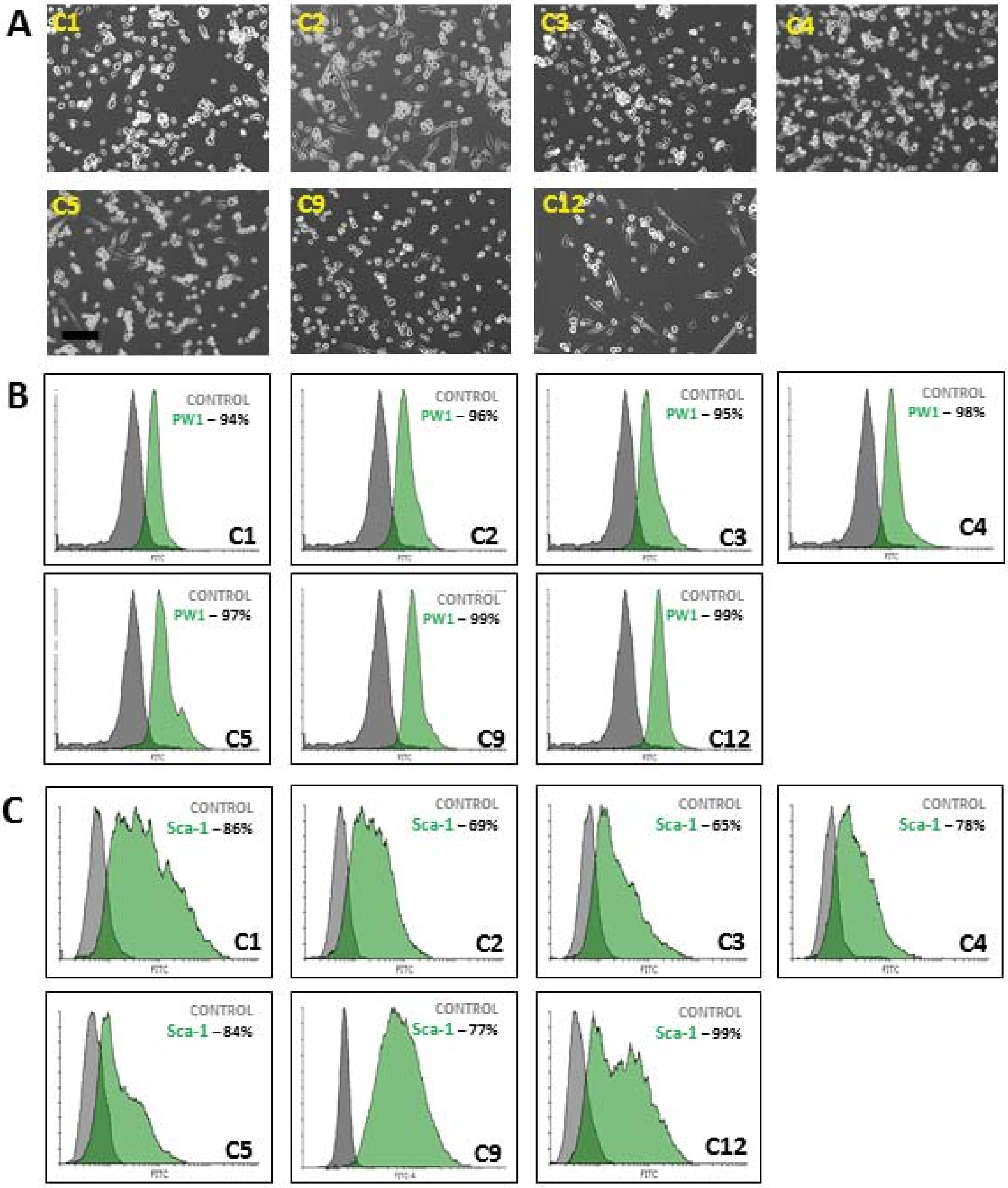

Supplementary Figure 2. Characterisation of clonal PICs. (**A**) Morphology of 7 PIC clones observed using light microscopy. Scale =200µm. (**B**) Flow cytometric analysis of PW1 expression in the 7 clonal PIC populations. (**C**) Flow cytometric analysis of Sca-1 expression in the 7 clonal PIC populations.
