# Supplementary material for: Skeletal muscle-derived interstitial progenitor cells (PICs) display stem cell properties, being clonogenic, self-renewing, and multi-potent in vitro and in vivo
Source: Stem Cell Res Ther. 2017 Jul 4;8:158. doi: 10.1186/s13287-017-0612-4 (PMC5496597; doi:10.1186/s13287-017-0612-4)
Supplement: Supplementary file 7 — Transcript analysis of clonal vs. bulk PICs. (A) qRT-PCR transcript analysis of PIC markers PW1, CD34, and Sca-1 in clonal PICs, compared to bulk PICs. (B) qRT-PCR transcript analysis of pluripotency markers Oct3/4, Sox2, and Nanog in clonal PICs compared to bulk PICs. Bars represent the mean transcript copy number normalised to GAPDH. Error bars represent the standard deviation of the mean; n = 3. (PDF 84 kb) [file 13287_2017_612_MOESM6_ESM.pdf]

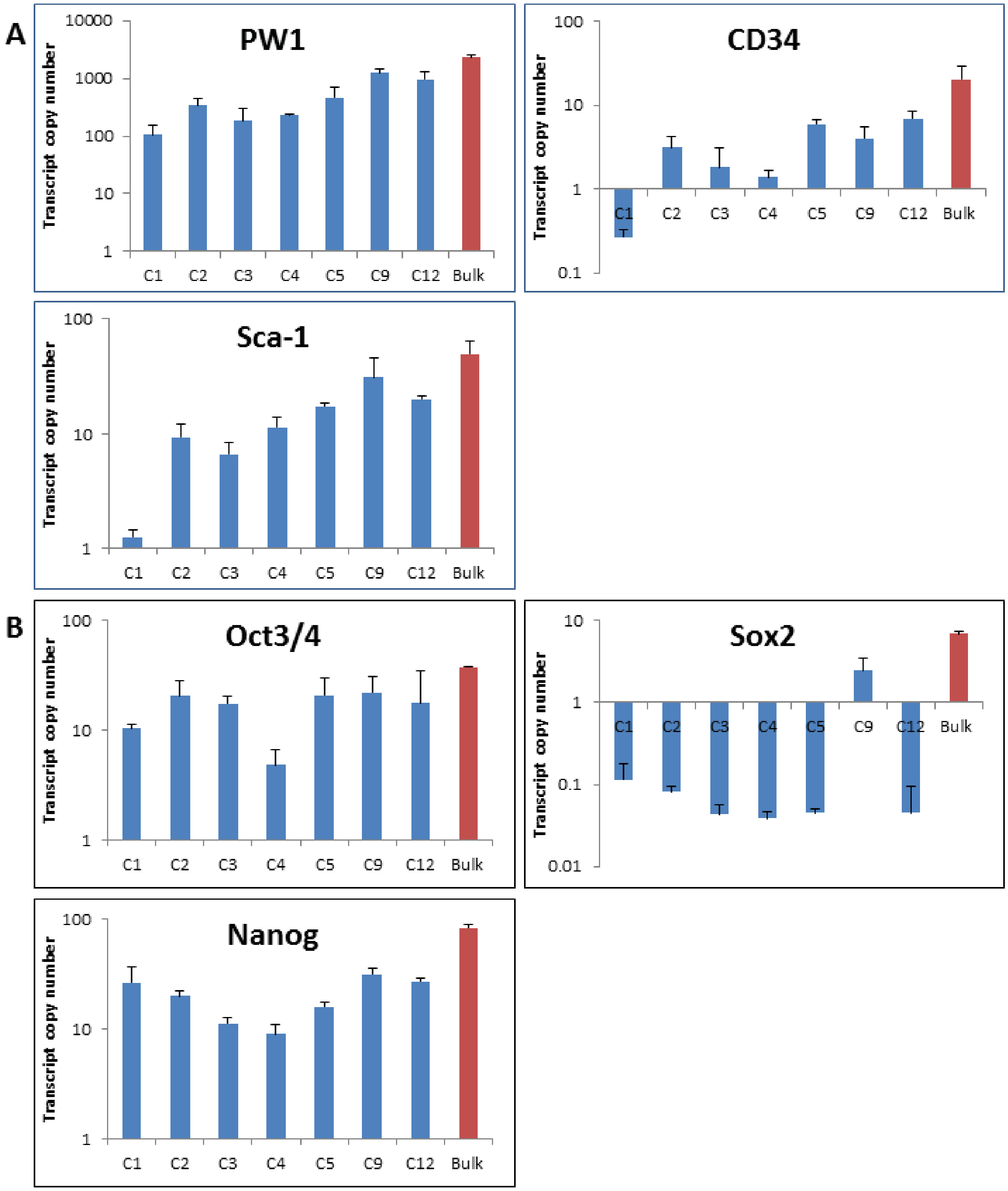

Supplementary Figure 3. Transcript analysis of clonal vs. bulk PICs. (**A**) qRT-PCR transcript analysis of PIC markers PW1, CD34 and Sca-1 in clonal PICs, compared to bulk PICs. (**B**) qRT-PCR transcript analysis of pluripotency markers Oct3/4, Sox2 and Nanog in clonal PICs compared to bulk PICs. Bars represent the mean transcript copy number normalised to GAPDH. Error bars represent the standard deviation of the mean, n=3.
