# Supplementary material for: Skeletal muscle-derived interstitial progenitor cells (PICs) display stem cell properties, being clonogenic, self-renewing, and multi-potent in vitro and in vivo
Source: Stem Cell Res Ther. 2017 Jul 4;8:158. doi: 10.1186/s13287-017-0612-4 (PMC5496597; doi:10.1186/s13287-017-0612-4)
Supplement: Supplementary file 8 — Phenotypic stability of C9 over 20 culture passages. Flow cytometric analysis at (A) P2 and (B) P20 quantifying expression of CD34, PDGFRα, CXCR4, PDGFRβ, c-kit, CD31, CD146, and NG2 in C9 PICs. (PDF 168 kb) [file 13287_2017_612_MOESM7_ESM.pdf]

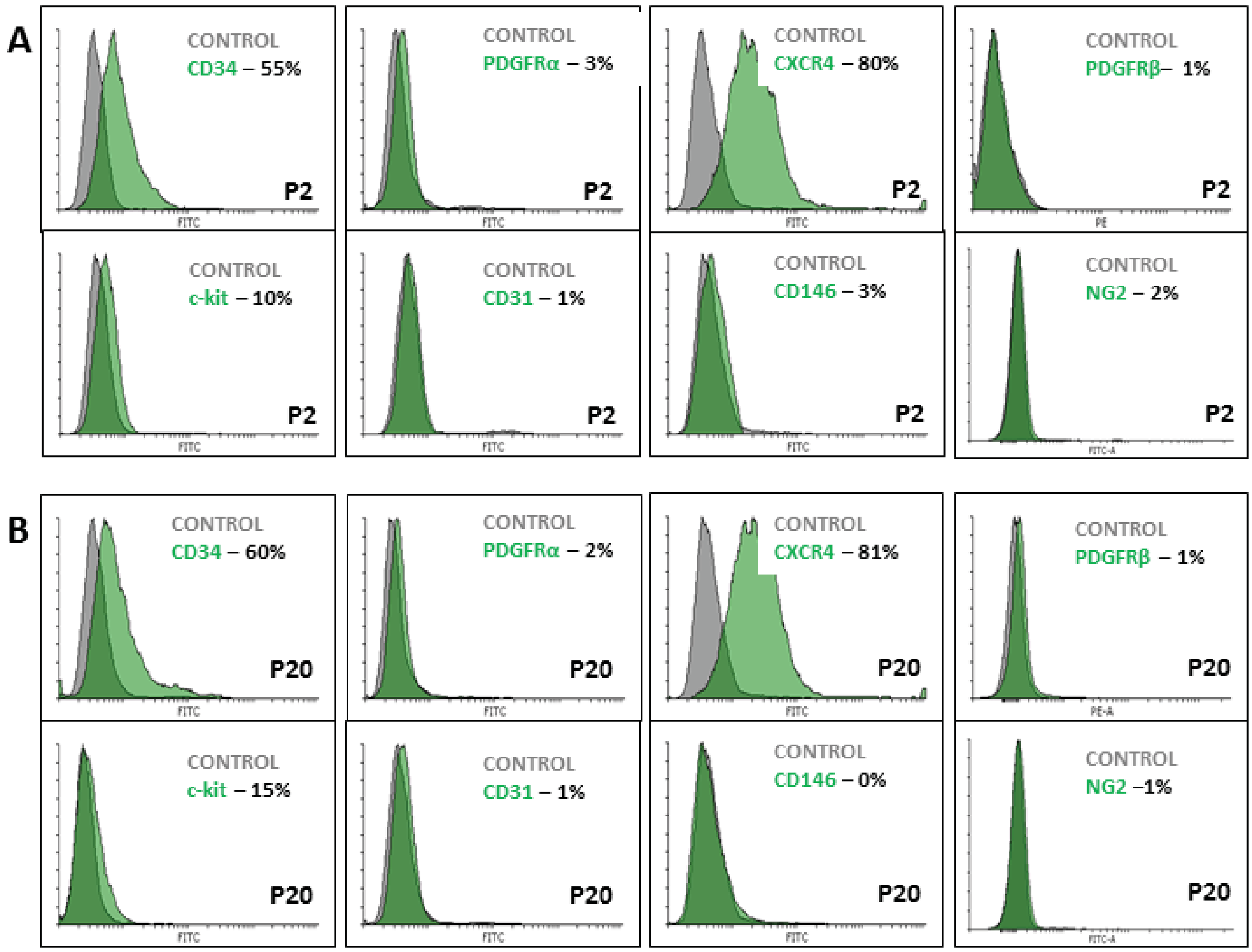

Supplementary Figure 4. Phenotypic stability of C9 over 20 culture passages. Flow cytometric analysis at (**A**) P2 and (**B**) P20 quantifying expression of CD34, PDGFRα, CXCR4, PDGFRβ, c-kit, CD31, CD146, NG2 in C9 PICs.
