# Supplementary material for: Skeletal muscle-derived interstitial progenitor cells (PICs) display stem cell properties, being clonogenic, self-renewing, and multi-potent in vitro and in vivo
Source: Stem Cell Res Ther. 2017 Jul 4;8:158. doi: 10.1186/s13287-017-0612-4 (PMC5496597; doi:10.1186/s13287-017-0612-4)
Supplement: Supplementary file 9 — PW1/Sca-1 flow cytometric analysis of bulk, clonal, and sub-clonal PICs. (A) Bulk expression of PW1 and Sca-1 at P3. (B) C9 clone expression of PW1 and Sca-1 at P2. (C) C9A sub-clone expression of PW1 and Sca-1 at P2. Plots are representative of: bulk, n = 1; clones, n = 7; sub-clones, n = 3. (PDF 145 kb) [file 13287_2017_612_MOESM8_ESM.pdf]

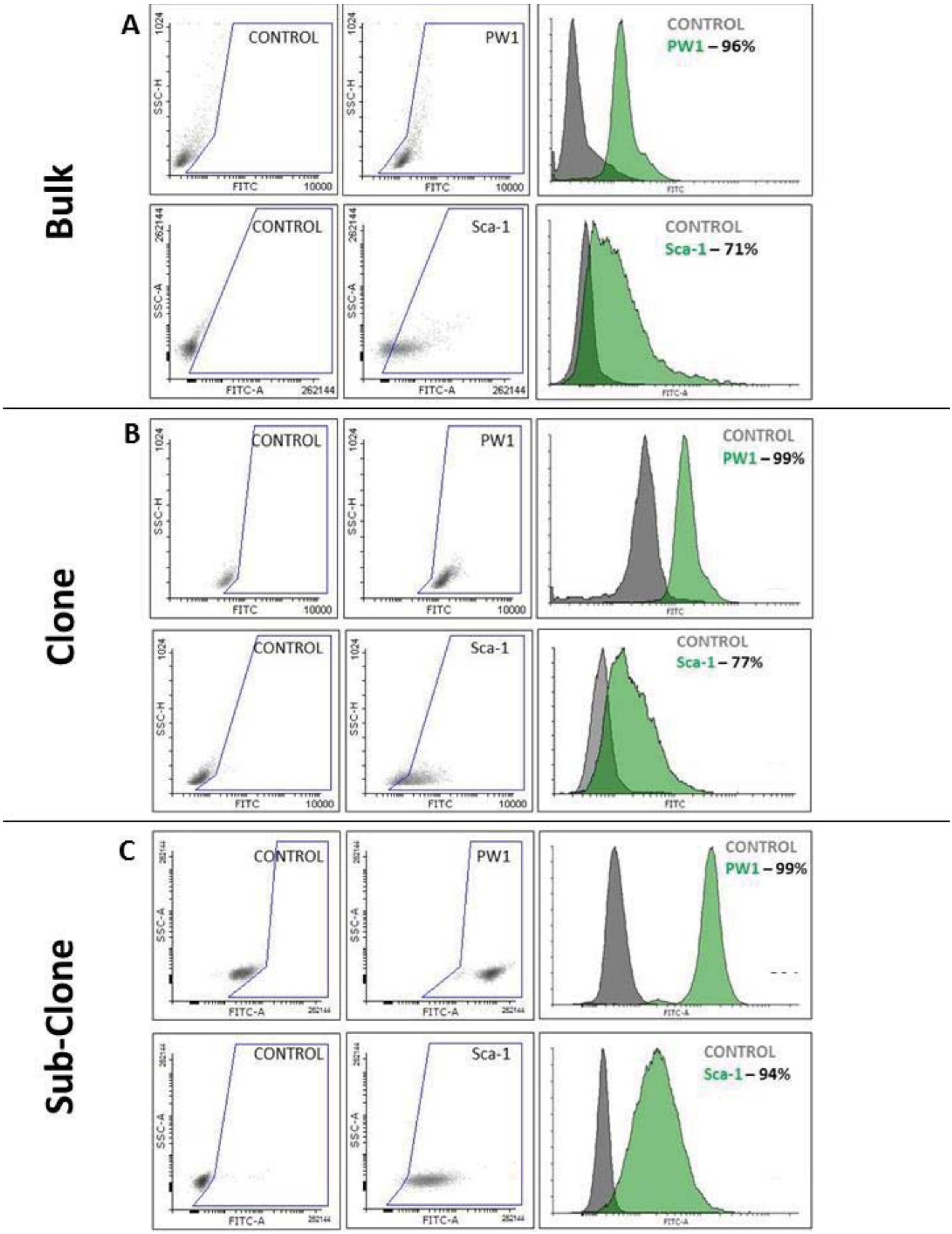

Supplementary Figure 5. PW1/Sca-1 flow cytometric analysis of bulk, clonal, sub-clonal PICs. (**A**) Bulk expression of PW1 and Sca-1 at P3. (**B**) C9 clone expression of PW1 and Sca-1 at P2. (**C**) C9A sub-clone expression of PW1 and Sca-1 at P2. Plots are representative of bulk, n=1; clones, n=7; sub-clones, n=3.
