# Supplementary material for: Skeletal muscle-derived interstitial progenitor cells (PICs) display stem cell properties, being clonogenic, self-renewing, and multi-potent in vitro and in vivo
Source: Stem Cell Res Ther. 2017 Jul 4;8:158. doi: 10.1186/s13287-017-0612-4 (PMC5496597; doi:10.1186/s13287-017-0612-4)
Supplement: Supplementary file 10 — Flow cytometry and transcript analysis of C9 sub-clones vs. C9 PICs. (A) qRT-PCR transcript analysis of sub-clones (C9A–C) compared to C9 PICs. Bars represent the mean relative expression normalised to GAPDH. Error bars represent the standard deviation of the mean; n = 3. (B) Flow cytometry histograms show consistent expression of Sca-1 and PW1 in sub-clones (C9A–C). (PDF 124 kb) [file 13287_2017_612_MOESM9_ESM.pdf]

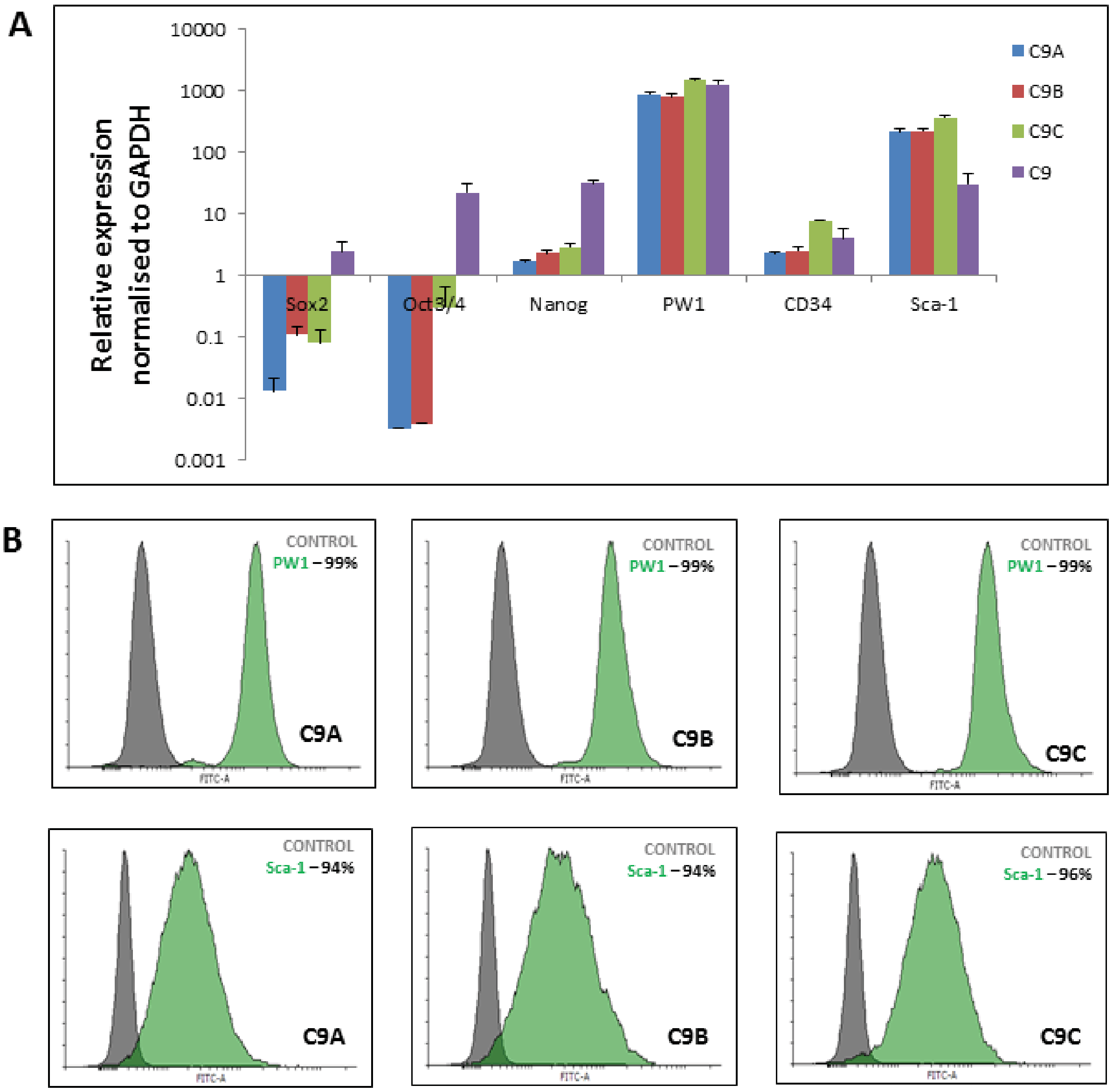

Supplementary Figure 6. Flow cytometry and transcript analysis of C9 sub-clones vs. C9 PICs. (**A**) qRT-PCR transcript analysis of sub-clones (C9A-C) compared to C9 PICs. Bars represent the mean relative expression normalised to GAPDH. Error bars represent the standard deviation of the mean, n=3. (**B**) Flow cytometry histograms show consistent expression of Sca-1 and PW1 in sub-clones (C9A-C).
