# Supplementary material for: Skeletal muscle-derived interstitial progenitor cells (PICs) display stem cell properties, being clonogenic, self-renewing, and multi-potent in vitro and in vivo
Source: Stem Cell Res Ther. 2017 Jul 4;8:158. doi: 10.1186/s13287-017-0612-4 (PMC5496597; doi:10.1186/s13287-017-0612-4)
Supplement: Supplementary file 11 — Transcript analysis of PICs differentiated into the three germ layers in vitro. (A–C) qRT-PCR analysis of the fold change increase in endothelial (A), hepatic (B), and neuronal (C) transcripts of differentiated C9 PICs, compared to undifferentiated PICs. (D) qRT-PCR analysis of myogenic, cardiomyogenic, endothelial, hepatic, and neuronal transcripts of undifferentiated C9 PICs. Bars represent the mean relative expression normalised to GAPDH. Error bars represent the standard deviation of the mean; n = 3. (PDF 82 kb) [file 13287_2017_612_MOESM11_ESM.pdf]

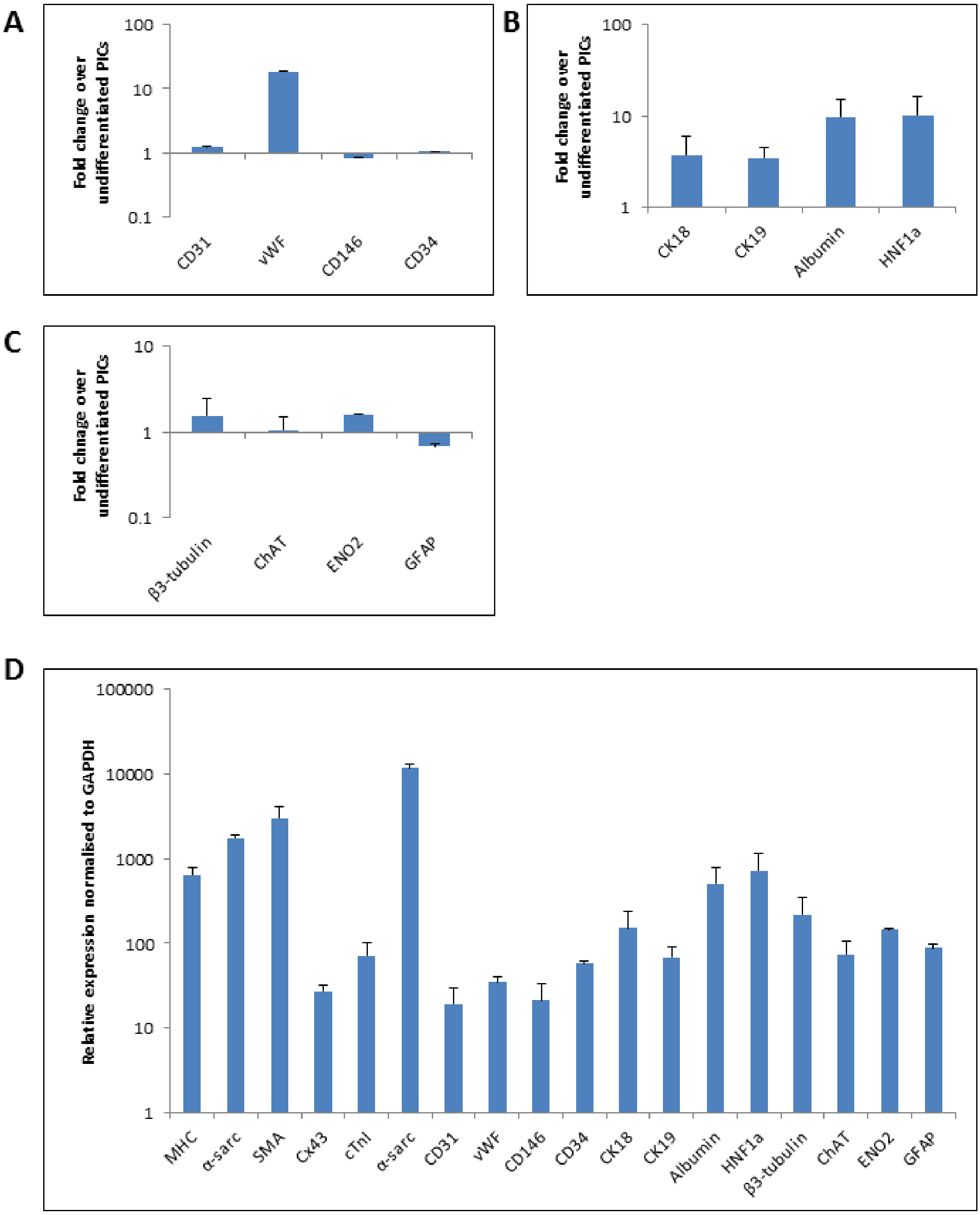

Supplementary Figure 7. Transcript analysis of PICs differentiated into the 3 germ layers *in vitro*. (**A-C**) qRT-PCR analysis of the fold change increase in endothelial (**A**), hepatic (**B**) and neuronal (**C**) transcripts of differentiated C9 PICs, compared to undifferentiated PICs. (**D**) qRT-PCR analysis of myogenic, cardiomyogenic, endothelial, hepatic, and neuronal transcripts of undifferentiated C9 PICs. Bars represent the mean relative expression normalised to GAPDH. Error bars represent the standard deviation of the mean, n=3.
