# Supplementary material for: Skeletal muscle-derived interstitial progenitor cells (PICs) display stem cell properties, being clonogenic, self-renewing, and multi-potent in vitro and in vivo
Source: Stem Cell Res Ther. 2017 Jul 4;8:158. doi: 10.1186/s13287-017-0612-4 (PMC5496597; doi:10.1186/s13287-017-0612-4)
Supplement: Supplementary file 12 — Multipotency of PICs in vivo. (A) Fluorescent microscope images of GFP-transduced C9 PICs. Scale bar = 200 μm. (B) Flow cytometric analysis of GFP expression in transduced C9 PICs (green histogram), compared to mock transduced cells (grey histogram). (C) Teratomas viewed on the kidney of mice after 4 weeks. No teratomas evident in SHAM (n = 2) or PIC-treated animals (n = 3). Teratoma formation observed in PIC/ESC (n = 3) and ESC-treated (n = 2) animals. (D) Variety of cell morphologies seen in teratomas, visualised by haematoxylin and eosin staining. Scale bar = 200 μm (top left) and 20 μm all other images. (E,F) GFP was detected in teratomas generated by GFP+ PIC/ESC-treated mice only and not in ESC-treated mice, shown by DAB staining (E) and immunofluorescence staining (F). Scale bar = 100 μm. (PDF 713 kb) [file 13287_2017_612_MOESM12_ESM.pdf]

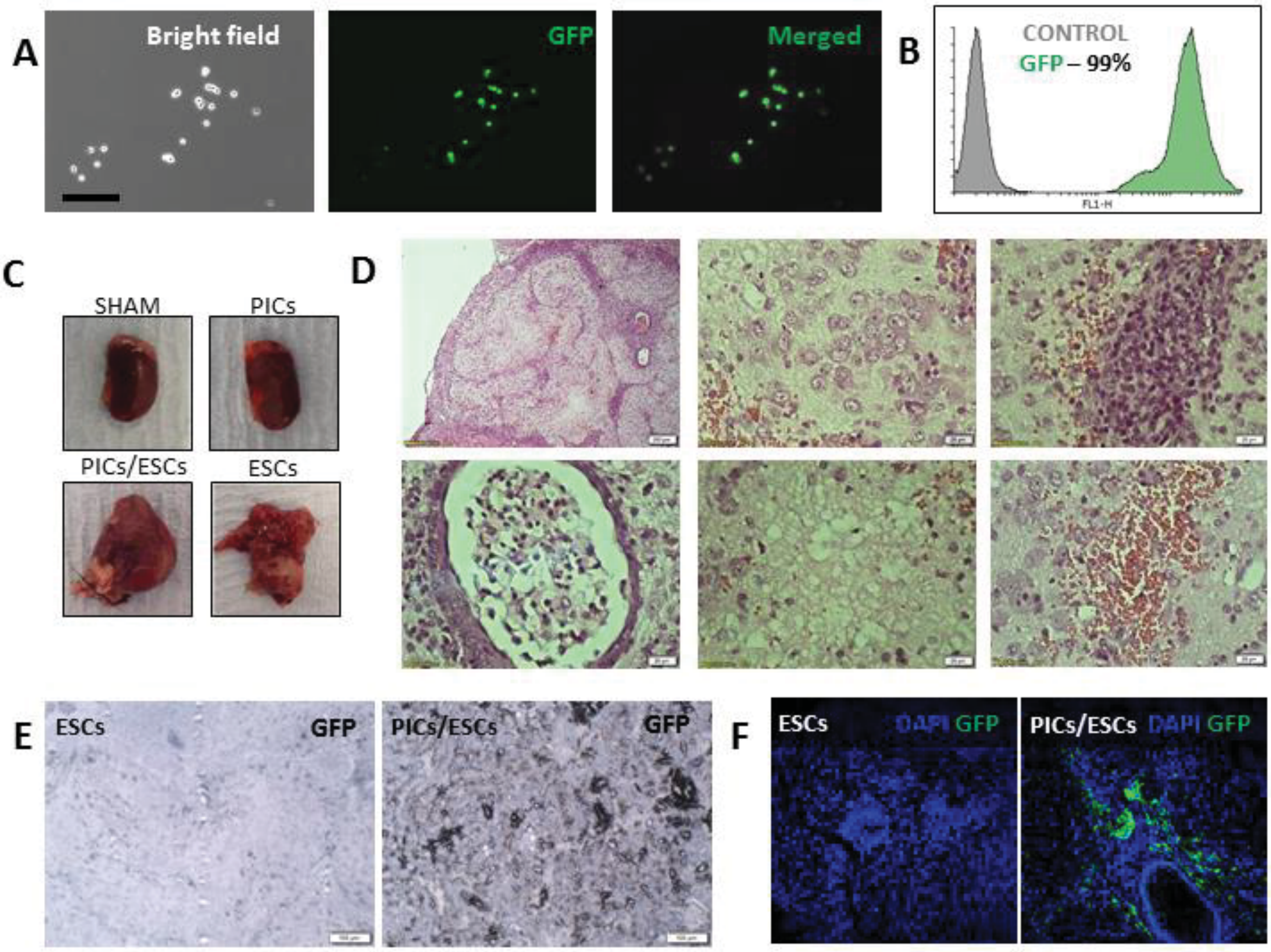

Supplementary Figure 8. Multipotency of PICs *in vivo*. (**A**) Fluorescent microscope images of GFP-transduced C9 PICs. Scale =200µm. (**B**) Flow cytometric analysis of GFP expression in transduced C9 PICs (green histogram), compared to mock transduced cells (grey histogram) (**C**) Teratomas viewed on the kidney of mice after 4 weeks. No teratomas evident in SHAM, (n=2) or PIC-treated animals (n=3). Teratoma formation observed in PIC/ESC, (n=3) and ESC-treated, (n=2) animals. (**D**) Variety of cell morphologies seen in teratomas; visualised by haematoxylin and eosin staining. Scale =200µm (top left) and 20µm all other images. (**E-F**) GFP was detected in teratomas generated by GFP^{pos} PIC/ESC-treated mice only and not in ESC-treated mice, shown by DAB staining (**E**) and immunofluorescence staining (**F**). Scale =100µm.
